# Dynamical Allocation of Cellular Resources as an Optimal Control Problem: Novel Insights into Microbial Growth Strategies

**DOI:** 10.1371/journal.pcbi.1004802

**Published:** 2016-03-09

**Authors:** Nils Giordano, Francis Mairet, Jean-Luc Gouzé, Johannes Geiselmann, Hidde de Jong

**Affiliations:** 1 Université Grenoble Alpes, Laboratoire Interdisciplinaire de Physique (CNRS UMR 5588), Saint Martin d’Hères, France; 2 Inria, Grenoble - Rhône-Alpes research centre, Montbonnot, Saint Ismier Cedex, France; 3 Inria, Sophia-Antipolis Méditerranée research centre, Sophia-Antipolis Cedex, France; Rice University, UNITED STATES

## Abstract

Microbial physiology exhibits growth laws that relate the macromolecular composition of the cell to the growth rate. Recent work has shown that these empirical regularities can be derived from coarse-grained models of resource allocation. While these studies focus on steady-state growth, such conditions are rarely found in natural habitats, where microorganisms are continually challenged by environmental fluctuations. The aim of this paper is to extend the study of microbial growth strategies to dynamical environments, using a self-replicator model. We formulate dynamical growth maximization as an optimal control problem that can be solved using Pontryagin’s Maximum Principle. We compare this theoretical gold standard with different possible implementations of growth control in bacterial cells. We find that simple control strategies enabling growth-rate maximization at steady state are suboptimal for transitions from one growth regime to another, for example when shifting bacterial cells to a medium supporting a higher growth rate. A near-optimal control strategy in dynamical conditions is shown to require information on several, rather than a single physiological variable. Interestingly, this strategy has structural analogies with the regulation of ribosomal protein synthesis by ppGpp in the enterobacterium *Escherichia coli*. It involves sensing a mismatch between precursor and ribosome concentrations, as well as the adjustment of ribosome synthesis in a switch-like manner. Our results show how the capability of regulatory systems to integrate information about several physiological variables is critical for optimizing growth in a changing environment.

## Author Summary

Microbial growth is the process by which cells sustain and reproduce themselves from available matter and energy. Strategies enabling microorganisms to optimize their growth rate have been extensively studied, but mostly in stable environments. Here, we build a coarse-grained model of microbial growth and use methods from optimal control theory to determine a resource allocation scheme that would lead to maximal biomass accumulation when the cells are dynamically shifted from one growth medium to another. We compare this optimal solution with several cellular implementations of growth control, based on the capacity of the cell to sense different physiological variables. We find that strategies maximizing growth in steady-state conditions perform quite differently in dynamical conditions. Moreover, the control strategy with performance close to the theoretical maximum exploits information of more than one physiological variable, suggesting that optimization of microbial growth in dynamical rather than steady environments requires broader sensory capacities. Interestingly, the ppGpp alarmone system in the enterobacterium *Escherichia coli*, known to play an important role in growth control, has structural similarities with the control strategy approaching the theoretical maximum. It senses a discrepancy between the concentrations of precursors and ribosomes, and adjusts ribosome synthesis in an on-off fashion. This suggests that *E. coli* is adapted for environments with intermittent, rapid changes in nutrient availability.

## Introduction

Microorganisms adapt their physiology to changes in nutrient availability in the environment. This involves changes in the expression of a large number of genes, encoding proteins with a variety of cellular functions, such as transporters for the uptake of nutrients, enzymes for the conversion of nutrients to energy and building blocks for macromolecules, the components of the transcriptional and translational machinery, and transcription factors to preferentially direct RNA polymerase to specific promoters [1, 2]. Fundamentally, the reorganization of gene expression in response to changes in environmental conditions is a resource allocation problem. It poses the question how microorganisms redistribute their protein synthesis capacity over different cellular functions when constrained by the changing environment.

The mechanisms responsible for resource allocation in microbial cells are usually assumed to have been optimized through evolution, so as to maximize the offspring of cells in their natural environment. How this general principle manifests itself on the level of cellular physiology is not straightforward though. Many studies have reasoned that growth-rate maximization provides a selective advantage to microorganisms, because it allows competitors to be outgrown when resources are scarce. Others have shown, however, that appropriate optimization criteria will depend on the structure of the environment and the ecosystem, as well as on the molecular properties of metabolic pathways [3–7]. For instance, in environments without competition for a shared resource, maximization of growth yield rather than growth rate is expected to provide a selective advantage. Although what counts as optimal is thus context-dependent, growth and evolution experiments in *Escherichia coli* have shown that in certain conditions bacterial metabolism is indeed geared towards growth-rate maximization [8–10].

For this reason, growth-rate maximization is a central hypothesis in a number of recent theoretical studies of resource allocation using coarse-grained models of the cell [11–13]. The models deliberately reduce the molecular complexity of regulatory networks so as to focus on generic explanatory principles [14]. Along these lines, Molenaar *et al*. developed a series of simple models of the microbial cell, taking into account that growth requires the synthesis of proteins playing a role in metabolism (transporters, enzymes) and gene expression (ribosomes), in varying proportions. Allocation parameters that maximize the growth rate were shown to account, at least in a qualitative way, for the variation of the amount of ribosomal protein as a fraction of total protein in different growth media, and for the occurrence of overflow metabolism above certain growth rates [11]. Using another coarse-grained model of the cell, centered on amino acid supply (metabolism) and demand (protein synthesis), Scott *et al*. derived empirical growth laws with linear relations between the ribosomal protein fraction and the growth rate, in conditions where the nutrient supply or demand are altered [12, 13]. In their model, maximization of growth rate requires maximization of amino acid flux and is achieved for a specific, unique value of the ribosomal protein fraction. Based on a structurally similar model, Maitra and Dill related optimal resource allocation to the basic constants of the metabolic and gene expression machinery, in particular energy efficiency [15].

The assumption of growth-rate maximization may lead to correct predictions in some situations, but ignores the regulatory mechanisms achieving resource allocation and therefore cannot provide a causal explanation of cellular behavior [16]. Several studies have used coarse-grained models to understand which control strategies microorganisms employ to achieve (optimal) resource allocation [13, 17, 18]. Scott *et al*. have shown that a robust feedforward control strategy, based on the sensing of the amino acid pool size and the corresponding adjustment of the fraction of ribosomes producing ribosomal proteins, allows the ribosomal protein fraction to be maintained close to its optimal value under a variety of growth conditions [13]. The authors suggest that this control strategy involves the signalling molecule ppGpp, in agreement with conclusions drawn from a recent kinetic model of the regulatory mechanisms achieving optimal adjustment of the ribosomal protein fraction [17]. Weiße *et al*. also developed a coarse-grained model of microbial growth based on resource allocation trade-offs [18]. Without including specific regulatory interactions, the model accounts for the above-mentioned bacterial growth laws, predicts host-circuit interactions in synthetic biology, and relates gene regulation to the nutrient composition of the medium.

The above studies consider resource allocation at steady state, where all intensive variables describing the growing microbial culture, in particular the concentrations of its molecular components, are constant (see [19] for a precise definition of steady-state growth and the closely related notions of balanced and exponential growth). This requires an environment to be stable over a long period of time. Such conditions can be achieved in the laboratory [20], but many microorganisms naturally experience frequently-changing conditions. For example, *E. coli* can cycle between two distinct habitats, the mammalian intestine and the earth’s surface (water, sediment, soil) [21]. The bacteria transit through different microenvironments in the intestinal system, where they encounter different mixes of sugars [22]. They are even more challenged in the open environment outside the host, with a greatly fluctuating availability of carbon and energy sources and a large variability in temperature, osmolarity, oxygen, and microbial communities [23, 24].

This situation motivates a dynamical perspective on microbial growth and resource allocation [25–28]. However, fundamental results like the growth laws uncovered for steady-state conditions are still lacking. In particular, extending the results reviewed above to dynamical conditions raises the following questions: Are control strategies that maximize steady-state growth also optimal in dynamical environments? If this is not the case, then which alternative strategies would be optimal for such conditions? And finally, how do these strategies compare with the regulatory mechanisms that have actually evolved in microorganisms?

The aim of this study is to address the above fundamental questions in a specific dynamical growth scenario, namely a transition between two steady states following an environmental perturbation. In particular, we consider the upshift of a microbial culture from a medium supporting growth at a low rate to a medium supporting growth at a high rate [28]. We develop a coarse-grained model of the cell, inspired by the self-replicator model of Molenaar *et al*. [11], and reformulate our questions in the context of optimal control theory [29] to identify control schemes maximizing biomass production over an interval of time, the dynamical equivalent of growth-rate maximization.

We show that Pontryagin’s Maximum Principle suggests that optimal resource allocation after a growth transition is achieved by a bang-bang-singular control law [29], a conjecture confirmed by direct numerical optimization. This optimal solution provides a gold standard against which possible control strategies of the cell can be compared. We consider simple strategies that drive the system to the steady state enabling growth at the maximal rate in the new medium, after the upshift. In a dynamical growth scenario, the strategy sensing the concentration of precursor metabolites emerges as the best candidate, consistent with the analysis of Scott *et al*. that feedforward activation of the rate of synthesis of ribosomal proteins, involving ppGpp-mediated sensing of the amino acid pool [30–32], is the key regulatory mechanism for growth control. It is possible, however, to define a strategy approaching the theoretical optimum even more closely by exploiting information on both the precursor concentration and the abundance of the gene expression machinery. Interestingly, a thorough analysis of the functioning of the ppGpp system, as described by a kinetic model of the synthesis and degradation of this signalling molecule, suggests similarities between our two-variable control strategy and the regulation of the transcription of ribosomal RNA by ppGpp [17].

The results presented here generalize the analysis of control strategies enabling optimal growth of microorganisms from steady-state to dynamical scenarios. The control strategies are formulated in the context of a coarse-grained model of resource allocation, based on minimal assumptions, that accounts for empirical growth laws at steady state. The analysis shows that during growth transitions, control strategies based on information of a single variable are outperformed by systems measuring several variables. This conclusion agrees with the intuition that, in dynamical environments, there may be an evolutionary pressure towards more elaborate sensory systems. From a methodological point of view, our study illustrates how optimal control theory can provide novel insights into complex biological phenomena [33].

## Results

### Self-replicator model of resource allocation

Resource allocation in bacteria involves the distribution of cellular resources (precursor metabolites and energy) over processes supporting maintenance and growth [1]. A simple modelling tool for analyzing resource allocation questions in a precise way are so-called self-replicator models. These models have a long history in various domains of chemistry, biology, physics, and computer science [34], and were recently put to use as an analytical tool in systems biology [11] (see also [35]). We will show that despite their simplicity, which make them tractable for mathematical analysis, self-replicator models are sufficiently expressive to account for empirical observations and make testable predictions.

**Fig 1:**
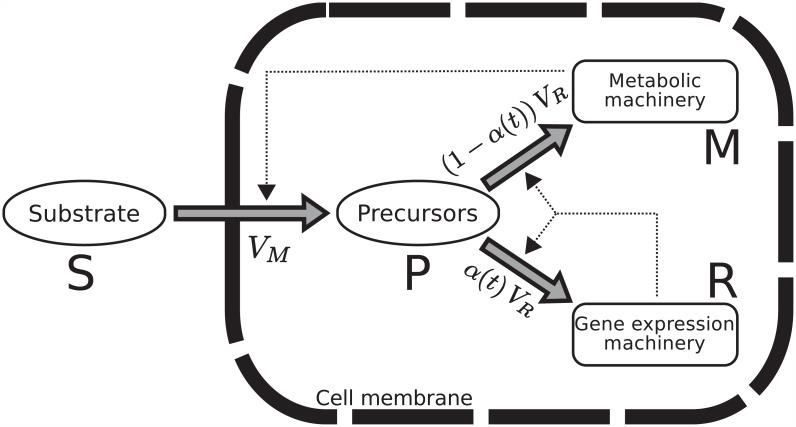
Self-replicator model of bacterial growth. External substrates *S* enter the cell and are transformed into precursors *P* through the action of the metabolic machinery *M*. The precursors are used by the gene expression machinery *R* to make the proteins composing both the metabolic machinery (transporters, enzymes,…) and the gene expression machinery itself (RNA polymerase, ribosomes,…). *α* (1 − *α*) is the mass proportion of precursors converted into *R* (*M*). Thick arrows denote reactions and thin, dashed arrows denote catalytic activities. The rate of synthesis of precursors and the rate of synthesis of proteins from precursors are denoted by *V*_*M*_ and *V*_*R*_, respectively.

Bearing in mind that the major constituents of the cell are macromolecules (DNA, RNA, proteins), produced from precursor metabolites, a fundamental resource allocation question is the following: How much of the cellular resources are invested in the making of new macromolecules (gene expression machinery) and how much in performing other functions, in particular producing metabolic enzymes involved in the uptake of nutrients and their conversion to precursor metabolites (metabolic machinery)? In order to address this question, we consider a self-replicating system composed of the gene expression machinery (*R*) and the metabolic machinery (*M*). The system, shown schematically in Fig 1, is thus defined by two macroreactions which are conveniently written as:

$$\begin{array}{cc} S & \overset{V_{M}}{⟶}P,\\ P & \overset{V_{R}}{⟶}\alpha R+\left(1-\alpha \right)M. \end{array}$$ (1)

The first reaction, catalyzed by *M*, converts external substrates (*S*) into precursor metabolites (*P*). The second reaction, catalyzed by *R*, converts precursors into macromolecules (*R* and *M*). The resource allocation parameter *α* ∈ [0, 1] defines the proportion of precursor mass used for making gene expression machinery as compared to metabolic machinery. We will interchangeably use the symbols *M*, *R*, *S*, and *P* for the components of the replicators themselves and their total mass [g]. We will denote the rates at which the macroreactions occur by *V*_*R*_ and *V*_*M*_ [g h^-1^].

The self-replicator system in Fig 1 is based on a number of simplifying assumptions. First, cell division is not explicitly modelled and replication should therefore be interpreted as the growth of (the mass of) a cell population. This amounts to the assumption that individual cells in a growing populations have the same macromolecular composition. Second, degradation of the macromolecules is ignored. In other words, we assume that macromolecules are stable and that their degradation rates are negligible with respect to the rates of other reactions in the system. Third, we consider only two classes of macromolecules (*R* and *M*). In particular, we do not assume that an irreducible mass fraction of the precursors is dedicated to cell maintenance [12]. The system could be easily extended to relax the above assumptions, but this would complicate the analysis of the model and obscure the points we want to make.

In what follows, it will be more convenient to describe the quantities in the system as intracellular concentrations rather than as the total mass in the cell population. To this end, we define the volume Vol [L] of the cell population as follows:

Vol=β(M+R), (2)

with *β* a conversion constant [L g^-1^] equal to the inverse of the cytoplasmic density. Dividing each variable *M*, *R*, and *P* by Vol yields the concentrations *m*, *r*, and *p* of metabolic enzymes, ribosomes and other components of the gene expression machinery, and precursor metabolites, respectively [g L^-1^]. Henceforth, these variables as well as Vol and *α* will be considered functions of time *t* [h].

The dynamics of the self-replicator in Fig 1 can be described by the following system of ordinary differential equations (see S1 Text for the derivation):

$$\frac{dp}{dt}=v_{M}\left(s,r\right)-v_{R}\left(p,r\right)\;\left(1+\beta \;p\right),$$ (3)

$$\frac{dr}{dt}=v_{R}\left(p,r\right)\;\left(\alpha \left(t\right)-\beta \;r\right),$$ (4)

where *s* [g L^-1^] denote the (extracellular) concentration of substrate. *v*_*M*_(*s*, *r*) [g L^-1^ h^-1^] and *v*_*R*_(*p*, *r*) [g L^-1^ h^-1^] denote the precursor synthesis rate and the macromolecule synthesis rate, respectively. The growth rate *μ* [h^-1^] of the replicator system is defined as the relative increase of the volume, and can be rewritten with Eqs 3 and 4 as proportional to the macromolecule synthesis rate (S1 Text):

$$\mu =\frac{1}{\text{Vol}}\frac{d\text{Vol}}{dt}=\frac{1}{M+R}\frac{d(M+R)}{dt}=\beta \;v_{R}\left(p,r\right).$$ (5)

The precursor concentration changes through the joint effect of the precursor synthesis rate *v*_*M*_(⋅), the macromolecule synthesis rate *v*_*R*_(⋅), and the rate of growth dilution (*β v*_*R*_(⋅)*p*). The change in concentration of ribosomes and other components of the gene expression machinery is the net effect of the ribosome synthesis rate (*α*(⋅)*v*_*R*_(⋅)) and the rate of growth dilution (*β v*_*R*_(⋅)*r*). Remark that it is not necessary to add an equation for *m* because it follows from Eq 2 that *r* + *m* = 1/*β*, and therefore *dm*/*dt* = −*dr*/*dt*.

We use Michaelis-Menten kinetics to define the synthesis rate of each reaction:

$$v_{M}\left(s,r\right)=k_{M}\;m\;\frac{s}{K_{M}+s}=k_{M}\;\left(1/\beta -r\right)\;\frac{s}{K_{M}+s},$$ (6)

$$v_{R}\left(p,r\right)=k_{R}\;r\;\frac{p}{K_{R}+p},$$ (7)

with rate constants *k*_*M*_, *k*_*R*_ [h^-1^] and half-saturation constants *K*_*M*_, *K*_*R*_ [g L^-1^]. Note that the rate of precursor synthesis is proportional to the concentration of the components of the metabolic machinery, while the macromolecule synthesis rate is proportional to the concentration of the components of the gene expression machinery. These catalytic effects correspond to the dashed arrows in Fig 1. The rate constant *k*_*M*_ depends both on the quality of the nutrients in the medium (higher *k*_*M*_ for a richer medium) and on the metabolic efficiency of the macroreaction converting the substrate into precursors (higher *k*_*M*_ for a more efficient reaction). For convenience, we henceforth assume that the environmental conditions do not change over the time-interval considered, either because *s* is constant or because *s* ≫ *K*_*M*_, corresponding to a situation in which the substrate is available in excess. In both cases, *e*_*M*_(*s*) = *k*_*M*_ *s*/(*K*_*M*_ + *s*) is approximately constant, so that we can write

$$v_{M}\left(r\right)=e_{M}\;\left(1/\beta -r\right).$$ (8)

The rate constant *k*_*R*_ characterizes the efficiency of the gene expression machinery, depending on the elongation rate of ribosomes, among other things. The ratio *p*/*K*_*R*_ is an indicator of the saturation of the gene expression machinery by precursors.

The system of Eqs 3 and 4 thus has four parameters (*e*_*M*_, *k*_*R*_, *K*_*R*_, *β*), one of which characterizes the input from the environment (*e*_*M*_). The order of magnitude of the parameters can be inferred from data in the literature, as explained in S2 Text. Below we use the following values for the parameters *e*_*M*_ = 3.6 h^-1^, *k*_*R*_ = 3.6 h^-1^, *K*_*R*_ = 1 g L^-1^, *β* = 0.003 L g^-1^ (S1 Table). However, it should be emphasized that the conclusions of this paper do not depend on the exact quantitative values of these parameters.

An interesting property of the model is that it is built on minimal assumptions, basically the two macroreactions and the definition of the volume as proportional to the total mass of macromolecules. Like in [11, 13, 25], these assumptions directly lead to the expression of the growth rate in Eq 5, without additional assumptions.

### Growth-rate maximization of the self-replicator reproduces bacterial growth laws

The nullcline for *r* is given by *r* = 0, *r* = *α*/*β*, and *p* = 0, while the nullcline for *p* is defined by

$$r=\frac{e_{M}}{\beta \;\left(e_{M}+k_{R}\;\frac{p}{K_{R}+p}\;\left(1+\beta p\right)\right)}.$$

**Fig 2:**
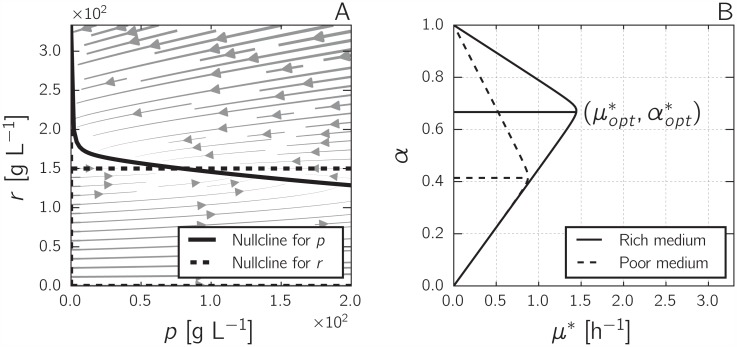
Analysis of self-replicator model of bacterial growth. *A:* Phase-plane analysis of the self-replicator model of Eqs 3 and 4. The nullclines for *p* and *r* are shown as solid and dashed curves, respectively. Parameter values are *e*_*M*_ = 3.6 h^-1^, *k*_*R*_ = 3.6 h^-1^, *K*_*R*_ = 1 g L^-1^, *β* = 0.003 L g^-1^, *α* = 0.45. *B:* Dependence of the growth rate at steady state μ* on the resource allocation parameter *α*, for two different environmental conditions (solid line, *e*_*M*_ = 4.76 h^-1^; dashed line, *e*_*M*_ = 1.57 h^-1^, other parameter values are *k*_*R*_ = 2.23 h^-1^, *K*_*R*_ = 1 g L^-1^, and *β* = 0.003 L g^-1^). The maximal growth rate is attained for a unique *α*, called $\alpha_{opt}^{*}$.

The nullclines define a single stable steady state (p*, r*) (Fig 2*A* and Methods). At this steady state, the growth rate is constant and denoted by μ*. The nullcline for *p* is defined by the environment *e*_*M*_. The nullcline for *r*, and thus the location of the steady state with the associated growth rate, are given by *α*. Fig 2*B* shows the dependency of the steady-state growth rate μ* on the resource allocation parameter *α*. As can be seen, μ* is maximal for a specific, unique value of *α*, which we denote $\alpha_{opt}^{*}$. That is, the model predicts that there is a single optimal way to divide the precursor flux over the synthesis of the gene expression machinery and the metabolic machinery. The same result, using a similar model, was obtained by Scott *et al*. [13]. The self-replicator model is simple enough to derive an algebraic expression for computing $\alpha_{opt}^{*}$ and the corresponding maximal growth rate $\mu_{opt}^{*}$ (Methods and S1 Text), which will simplify analysis of the system in later sections.

**Fig 3:**
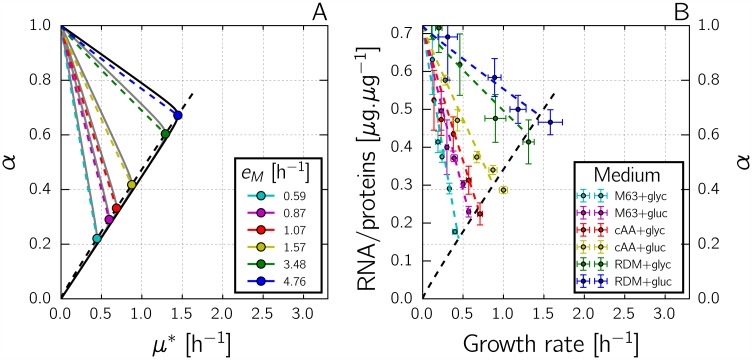
Self-replicator model accounts for bacterial growth laws. *A:* Predicted quasi-linear relation between the maximal growth rate $\mu_{opt}^{*}$ and the corresponding optimal resource allocation $\alpha_{opt}^{*}$, for different values of *e*_*M*_ (different colors). The colored dots indicate $\alpha_{opt}^{*}$ and $\mu_{opt}^{*}$ for *k*_*R*_ = 2.23 h^-1^ and different *e*_*M*_, and the dashed black line the relation for all intermediate values of *e*_*M*_. The dashed colored lines indicate the relation between $\alpha_{opt}^{*}$ and $\mu_{opt}^{*}$ obtained when, for a given value of *e*_*M*_, the value of *k*_*R*_ is decreased (lower *k*_*R*_ leads to lower $\mu_{opt}^{*}$). The solid grey curves correspond to (μ*, *α*)-profiles like those shown in Fig 2*B*. *B:* Measured relation between the total RNA/protein mass ratio and the growth rate, in different growth media with different doses of a translation inhibitor (data from [12]). For each medium, indicated by a color, five different concentrations of inhibitor were used (higher dose leads to lower growth rate). Growth-medium compositions are given in the original publication and error bars represent standard deviations. The dashed black and colored lines are the same as in panel *A*, indicating the good quantitative correspondence between model predictions and experimental data for the chosen parameter values, obtained by fitting the model to the data points (see Methods for details).

In order to validate the model, we verified that it can account for data on the macromolecular composition of *E. coli* at steady state [12]. When optimizing *α* for different values of *e*_*M*_ (assuming cells attain maximal growth), the model predicts a relation between $\alpha_{opt}^{*}$ and $\mu_{opt}^{*}$ (colored dots and black dashed line in Fig 3*A*) that is quasi-linear for high growth rates. We compared this prediction with the results of experiments where the relation between the growth rate and the mass ratio of total RNA and protein was determined in different growth media (Fig 3*B*). In the framework of our model, different media correspond to different values of *e*_*M*_, and different total RNA/protein mass ratios to different values of *α* (up to a conversion factor), allowing a direct comparison of the model predictions in Fig 3*A* with the data in Fig 3*B* (see Methods). As can be seen, the model is able to account for the observed quasi-linear relation between the growth rate and the total mass ratio of RNA and protein. Moreover, for realistic values of *k*_*R*_ and *e*_*M*_, a good quantitative fit is obtained (Methods and S1 Table).

The data from Scott *et al*. also reveal a second apparently linear relation between the growth rate and the total RNA/protein mass ratio. This relation is obtained when varying, in the same growth medium, the efficiency of protein synthesis by adding different doses of an inhibitor of translation (chloramphenicol) [12]. Using the model, we computed $\alpha_{opt}^{*}$ and $\mu_{opt}^{*}$, for constant environment *e*_*M*_ and different values of the efficiency of protein synthesis *k*_*R*_ (dashed colored lines in Fig 3*A*). As can be seen in Fig 3*B*, the model also captures the second linear relation in the data.

We conclude that the self-replicator model is able to reproduce known observations of resource allocation in bacteria, so-called growth laws [12]. The model is similar to a model recently proposed by Scott *et al*. [13]. Contrary to the latter model, the translation rate is not assumed to be constant in the self-replicator model, but rather depends on precursor abundance, as proposed by the same authors in [36].

The above analysis of bacterial growth has two major limitations. First, the predictions of optimal resource allocation (the value of *α* leading to the maximal growth rate) hold at steady state, for a constant environment, whereas most bacteria are not expected to encounter such conditions outside the laboratory. An allocation of resources that is optimal for steady-state growth and constant over time may not be optimal in dynamical growth conditions. Second, while it predicts which value of *α* is optimal at steady state, the model says nothing about the strategies that could be used to control resource allocation and set *α* to its optimal value. In other words, how could bacterial cells use sensors of changes in their internal state and the environment to optimally adjust *α*? In what follows, we will address the above two questions, after having given a precise statement of the problem of optimal resource allocation in a dynamical environment in the next section.

### Biomass maximization as an optimal control problem

A self-replicator at steady state accumulates biomass according to Vol(0) *e*^μ* *t*^, *t* ∈ [0, *τ*], when μ* is the growth rate at steady state. The accumulation of biomass is obviously maximal when the growth rate is maximal ($\mu^{*}=\mu_{opt}^{*}$). In dynamical conditions, the growth rate is not constant and biomass accumulation is described more generally by:

$$\frac{d\text{Vol}}{dt}=\mu \left(t\right)\;\text{Vol}.$$

In other words, when integrating over the time interval [0, *τ*]:

$$\ln\left(\frac{\text{Vol}(\tau )}{\text{Vol}(0)}\right)=\int_{0}^{\tau }\mu \left(t\right)\;dt.$$ (9)

Since the logarithm is an increasing function, maximizing the biomass produced over [0, *τ*] requires maximization of the right-hand side of the equation.

In a changing environment, maximization of the integral in Eq 9 will generally require the optimal value of *α* to be a function of time instead of a specific constant value. This dynamical resource allocation problem can be formulated in a more precise way using concepts from optimal control theory [29]. Let *J* be the objective function

$$J\left(\alpha \right)=\int_{0}^{\tau }\mu \left(t\right)\;dt=\int_{0}^{\tau }\beta \;v_{R}\left(p,r\right)\;dt,$$

where *α* : ℝ^+^ → [0, 1] is a time-dependent function. The time evolution of *p* and *r* is determined by the self-replicator model of Eqs 3 and 4, and *p* and *r* thus depend on *e*_*M*_ and *α*. Moreover, let $U=\{\alpha :R^{+}\to \left[0,1\right]\}$ be the set of admissible controls. The optimal dynamical control problem then consists in finding the time-varying function *α*_*opt*_(*t*) that maximizes *J*(*α*) over the time-interval [0, *τ*]:

$$\alpha_{opt}=\arg\max_{\alpha \in U}\;J\left(\alpha \right).$$ (10)

In what follows, we will simplify the above problem by considering that the environment changes in a step-wise fashion at *t* = 0, but remains constant over the time-interval [0, *τ*], that is, *e*_*M*_(*t*) = *e*_*M*_. More specifically, we focus on the case of a nutrient upshift, corresponding to a step-wise increase of *e*_*M*_. This upshift scenario corresponds to classical experiments in bacterial physiology [37–39], reviewed in [28], and is frequently encountered in the life cycle of a microorganism [1]. Notice that more complex environments can be approximated by a sequence of step-wise nutrient upshifts and downshifts.

### Solution of the optimal control problem

Optimal dynamical control problems for two-dimensional nonlinear dynamical systems, like the problem of Eq 10, are generally difficult to solve. However, we will show that the class of functions to which *α*_*opt*_ belongs can be identified, and we will use numerical optimization to identify a particular *α*_*opt*_ maximizing *J*.

As a preliminary step, in order to simplify the analysis, the variables in the self-replicator model of Eqs 3 and 4 are made nondimensional, by defining $\hat{t}=k_{R}\;t$, $\hat{p}=\beta \;p$, and $\hat{r}=\beta \;r$. This leads to the following ODE system:

$$\frac{d\hat{p}}{d\hat{t}}=\left(1-\hat{r}\right)\;E_{M}-\left(1+\hat{p}\right)\;\hat{r}\;\frac{\hat{p}}{K+\hat{p}},$$ (11)

$$\frac{d\hat{r}}{d\hat{t}}=\hat{r}\frac{\hat{p}}{K+\hat{p}}\;\left(\alpha \left(\hat{t}\right)-\hat{r}\right),$$ (12)

where *K* = *β K*_*R*_ and *E*_*M*_ = *e*_*M*_/*k*_*R*_. The nondimensional growth rate is given by:

$$\hat{\mu }=\frac{\mu }{k_{R}}=\frac{\hat{p}}{K+\hat{p}}\;\hat{r}.$$ (13)

Notice that the nondimensionalized system depends on a single parameter *K*, in addition to the constant environment *E*_*M*_, which functions as an input to the system.

**Fig 4:**
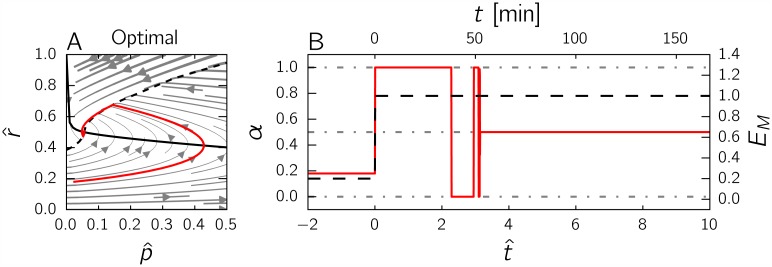
Optimal control of the self-replicator during a nutrient upshift. *A:* Optimal trajectory in the phase plane for the nondimensionalized model of Eqs 11 and 12, with streamlines. The optimal trajectory is shown as a solid, red curve. The solid, black curve represents the $\hat{p}$-nullcline. The dashed, black curve is the switching curve $\varphi (\hat{p})$. The optimal solution was obtained by numerical optimization using bocop [41] (see Methods for details), using the parameter values *E*_*M*_ = 1 and *K* = 0.003, and starting from the initial state (0.024, 0.18) at *t* = 0 (optimal steady state for *E*_*M*_ = 0.2). *B:* Time evolution of the control variable *α*_*opt*_(⋅) (thick, red line) and the environment *E*_*M*_ (dashed, black line).

Analysis of the nondimensionalized system allows a number of properties of the solution of the optimal control problem of Eq 10 to be derived (Methods and S3 Text). First, by applying a version of the well-known Pontryagin Maximum Principle [40], we can prove that the optimal solution is obtained for an alternating sequence of *α*(⋅) = 0 and *α*(⋅) = 1, possibly ending with an intermediate value of *α*(⋅), corresponding to the optimal steady state $\left(\hat{p}\left(t\right),\hat{r}\left(t\right)\right)=\left({\hat{p}}_{opt}^{*},{\hat{r}}_{opt}^{*}\right)$, that is, the steady state leading to the optimal growth rate ${\hat{\mu }}_{opt}^{*}$ in the post-upshift environment *E*_*M*_. Second, if the optimal solution reaches the optimal steady state for the new environment, then it does so after an infinite number of switches of *α*(⋅) between 0 and 1. Third, this switching behavior is characterized by a so-called switching curve $\hat{r}=\varphi \left(\hat{p}\right)$ in the $\left(\hat{p},\hat{r}\right)$-plane, which passes through $\left({\hat{p}}_{opt}^{*},{\hat{r}}_{opt}^{*}\right)$. The switching curve divides the phase plane into two regions, such that *α*(⋅) switches to 0 when the system is in the region above *φ* and to 1 when the system is below *φ* (black dashed curve in Fig 4*A*).

In line with these results, we conjecture that the optimal solution consists in a switching transient towards the optimal steady state for the new environment, and remains at this steady state until the next environmental change. Such a solution is known as a bang-bang-singular solution in the control theory literature [29]. Formally, the solution of Eq 10 can be described as

$$\alpha_{opt}\left(\hat{t}\right)=\left\{\begin{array}{c} 0,\;\text{if}\;\hat{r}\left(\hat{t}\right)>\varphi \left(\hat{p}\left(\hat{t}\right)\right),\\ 1,\;\text{if}\;\hat{r}\left(\hat{t}\right)<\varphi \left(\hat{p}\left(\hat{t}\right)\right),\\ \alpha_{opt}^{*},\;\text{if}\;\left(\hat{p}\left(\hat{t}\right),\hat{r}\left(\hat{t}\right)\right)=\left({\hat{p}}_{opt}^{*},{\hat{r}}_{opt}^{*}\right). \end{array}\right.$$ (14)

Notice that the optimal solution involves dynamical feedback from the state of the system to the control variable *α*(⋅), and is therefore an instance of closed-loop optimization [29].

The optimal control problem of Eq 10 was also solved numerically by a direct method using the bocop software [41] (see Methods for details). A time discretization allows the problem to be transformed into a nonlinear optimization problem solved here by interior point techniques. The optimal trajectories obtained numerically confirm our conjecture that the optimal control is bang-bang-singular. An example solution, obtained by numerical optimization is shown in Fig 4. At time $\hat{t}=0$, *E*_*M*_ jumps from a low to a high value, corresponding to a nutrient upshift (dashed black line in Fig 4B). The optimal solution *α*_*opt*_ consists of a sequence of switches between *α* = 1, corresponding to maximal synthesis of the gene expression machinery, and *α* = 0, corresponding to maximal synthesis of the metabolic machinery, until $\left({\hat{p}}_{opt}^{*},{\hat{r}}_{opt}^{*}\right)$ is reached. *α* is then set to $\alpha_{opt}^{*}$, the value leading to the maximum growth rate in the new medium (here 0.5, for *E*_*M*_ = 1). The sequence of switches of *α* in Fig 4*B* corresponds to successive crossings of the switching curve in Fig 4*A*. In particular, the switch just after $\hat{t}=2$ corresponds to the first crossing of the switching curve; the subsequent switches accumulate around the steady state and are therefore difficult to identify in the plot.

What is the biological relevance of the bang-bang-singular solution maximizing growth of the bacterial self-replicator? In order to answer this question, we will investigate in the next two sections the different ways in which microorganisms could implement or have been shown to implement feedback growth control by sensing the environment and cellular physiology. Although the idealized solution proposed by optimal control theory will obviously not be found in nature, actual control strategies may produce solutions that are close. The optimal solution can thus be used as a gold standard, a benchmark for comparing actual control strategies.

### Simple feedback control strategies: exploiting information on nutrients or precursors

The control strategies that microbial cells have evolved to bring resource allocation in line with changes in the environment involve a variety of molecular mechanisms [42]. These mechanisms are responsible for sensing the environment and the physiological state of the cell, as well as for adjusting the expression of genes that encode components of the transcriptional and translational machinery, enzymes, transporters, and proteins with other metabolic functions.

**Fig 5:**
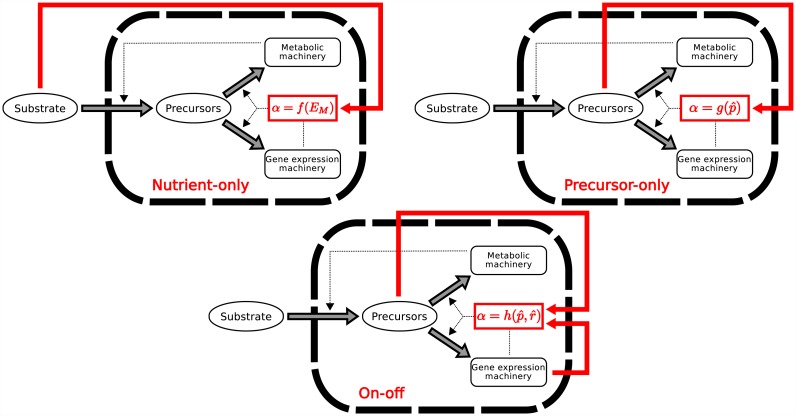
Alternative strategies for controlling the self-replicator of bacterial growth. The feedback control strategies, shown in red and superposed on the self-replicator of Fig 1, exploit information on system variables and the environment to adjust the value of *α*, and thus the relative allocation of resources to the metabolic machinery and gene expression machinery.

In the framework of the self-replicator model of bacterial growth, control strategies take the form of feedback control laws mapping the value of system variables to a value of the control variable *α*(⋅). In this section, we explore two such strategies, the first exploiting information on the quality and quantity of substrate present in the environment, as reflected in the value of *E*_*M*_, and the second using information on the precursor concentration $\hat{p}$. The feedback control strategies are graphically displayed in Fig 5, as an extension of the self-replicator of Fig 1. We pose a number of mathematical constraints on the feedback control strategies considered below. First, we require the control laws to be functions of the variables of the self-replicator but not involve derivatives or integrals of these variables. Second, for a constant environment *E*_*M*_, the control strategies must drive the system to a unique stable and non-trivial steady state, enabling a non-zero growth rate. Third, this steady state must equal the optimal steady state for that environment, given by $\left({\hat{p}}_{opt}^{*},{\hat{r}}_{opt}^{*}\right)$.

The first control strategy is defined by the function *f* : ℝ^+^ → [0, 1], mapping *E*_*M*_ to *α*:

$$\alpha =f(E_{M}).$$ (15)

Notice that *α* is constant because *E*_*M*_ is fixed to the value defining the new environment after the upshift. What would be an appropriate choice for *f*? An advantage of the self-replicator model is that the optimal allocation at steady state can be explicitly formulated as a function of *E*_*M*_ (Eq 22 in Methods, with derivation in S1 Text). This function is the unique function satisfying all of the above criteria (S1 Text). S1 Fig plots *f* and shows that it is conveniently approximated by a Michaelis-Menten function, *i.e*.,

$$\alpha \left(\cdot \right)=\frac{E_{M}}{E_{M}+K_{mE}},$$ (16)

with the dimensionless half-saturation constant *K*_*mE*_. The interest of the approximation is that it demonstrates that the control strategy can be described by a simple and ubiquitous response curve in biochemical kinetics.

As an example of a regulatory system resembling the above control strategy consider the phosphotransferase system responsible for the uptake of glucose, the preferred substrate of *E. coli* [43]. In the presence of glucose, the EIIA^Glc^ component of the phosphotransferase system is mostly unphosphorylated, since the phosphate groups are used for the conversion of extracellular glucose to intracellular glucose-6-phosphate. When glucose disappears from the medium, however, the glucose uptake rate decreases and, correspondingly, the phosphorylated fraction of EIIA^Glc^ increases. The phosphorylation state of EIIA^Glc^ thus provides an indirect read-out of glucose availability. In response to this signal, a variety of metabolic processes are upregulated or downregulated, notably involving the signalling molecule cAMP which activates the pleiotropic transcription factor Crp [43, 44].

**Fig 6:**
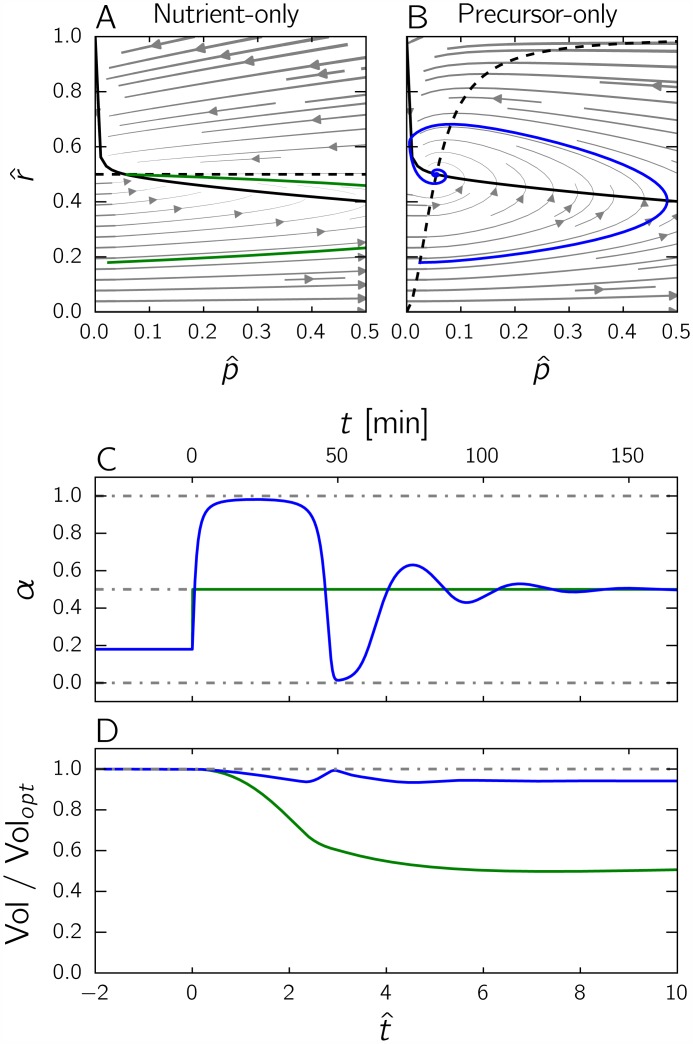
Comparison of the performance of the nutrient-only and precursor-only strategies after a nutrient upshift. *A:* Trajectory in the phase plane for the nutrient-only strategy (green curve). The solid, black curve represents the $\hat{p}$-nullcline. The dashed, black curve is the $\hat{r}$-nullcline. The solution is obtained by numerical simulation of the system of Eqs 11 and 12, supplemented with *α* = *f*(*E*_*M*_) as specified by Eq 27 in the *Methods* section and plotted in S1 Fig. The initial state corresponds to the steady state attained for an environment given by 0.2*E*_*M*_. While converging to the new steady state after the upshift, the precursor concentration makes a large overshoot. *B:* As above, but for the precursor-only strategy. The feedback control strategy is now defined by $\alpha =g(\hat{p})$ as specified by Eq 28 in the Methods section and plotted in S1 Fig. The solution trajectory (blue curve) exhibits a lower overshoot. *C:* Evolution of the control variable *α*(⋅) as a function of time, for each of the above two strategies. Notice that in the nutrient-only strategy *α*(⋅) immediately jumps to the optimal value for the post-upshift steady state (green curve), whereas in the precursor-only strategy it depends on the (time-varying) precursor concentration (blue curve). *D:* Evolution of the ratio Vol/Vol_*opt*_ as a function of time, where Vol is the volume of the self-replicator and Vol_*opt*_ the volume of the same replicator following the optimal strategy shown in Fig 4. In all of the above simulations, the parameter values *E*_*M*_ = 1 and *K* = 0.003 were used.

How does the control strategy of Eq 15, which we call a nutrient-only strategy, perform in comparison with the optimal solution derived in the previous section? That is, how much biomass does this strategy produce compared with the maximal amount of biomass that can theoretically be obtained after a nutrient upshift? In order to answer these questions, we simulated the response to a sudden upshift of the self-replicator of Eqs 11 and 12 controlled by the nutrient-only strategy of Eq 15. The results are shown in Fig 6. Panel *A* shows the trajectory of the controlled self-replicator system and panel *D* plots the evolution of the amount of biomass as a fraction of the amount of biomass produced by the optimal strategy. While the system does reach the steady state that is optimal for *E*_*M*_, the nutrient-only strategy has poor performance in the transient phase immediately following the nutrient upshift. As can be seen from the solution trajectory in Fig 6*A*, fixing *α* to the value that enables optimal growth at steady state leads to a huge transient overshoot of the precursor concentration. The overshoot reveals that resource allocation is initially suboptimal, with too many resources invested in the metabolic machinery at the expense of the gene expression machinery. This causes a transiently suboptimal growth rate, leading to lower biomass accumulation (Eq 9).

One way to avoid the transient precursor imbalance observed in Fig 6*A* would be to exploit information on the precursor concentration in the control strategy. The second strategy considered here, which we label a precursor-only strategy, does exactly this: it involves a feedback control law *g* : ℝ^+^ → [0, 1] mapping $\hat{p}$ to *α*:

$$\alpha =g(\hat{p}).$$ (17)

Since $\hat{p}$ will vary during the upshift experiment, *α* is not constant, contrary to the nutrient-only strategy above. In the Methods section, we present a function *g* satisfying the requirements listed in the beginning of this section, in particular that the system converge to a stable steady state ensuring maximal growth in the new environment. Moreover, we show that any other choice for *g* leads to lower biomass production. The function is plotted in S1 Fig, and as shown in the same panel, is conveniently approximated by a Hill function with cooperativity coefficient 2:

$$\alpha \left(\cdot \right)=\frac{{\hat{p}}^{2}}{{\hat{p}}^{2}+{K_{mp}}^{2}},$$ (18)

where *K*_*mp*_ is a dimensionless half-saturation constant.

While converging to the same steady state, this second strategy, which we will refer to as the precursor-only strategy, performs much better than the nutrient-only strategy after an upshift, as shown in Fig 6. We simulated the response to a nutrient upshift of the self-replicator of Eqs 11 and 12 with the precursor-only strategy of Eq 17. The relative biomass increases by 51% and reaches 94% of the biomass produced by the optimal control strategy (the theoretical maximum). The precursor-only strategy notably avoids the inefficient transient accumulation of precursors directly after the nutrient upshift, by alternatingly investing more resources in gene expression (consumption of precursors) and metabolism (production of precursors). In this respect, the oscillatory time profile of *α* (Fig 6*C*) is somewhat reminiscent of the bang-bang-singular control in the solution of the optimal control problem (Fig 4*B*).

Both strategies, nutrient-only and precursor-only, drive the self-replicator towards the same steady state. Whereas the two strategies are thus indistinguishable when the analysis is restricted to steady state, the precursor-only strategy is shown to perform much better in a dynamical upshift scenario, in the sense that the biomass produced is much closer to that produced by the optimal strategy. Several authors have concluded that control strategies based on precursor sensing are key for maintaining optimal growth at steady state. Scott *et al*. argue that a strategy similar to the precursor-only approach above allows robust control of amino acid supply and demand, resulting in optimal steady-state growth over a range of nutrient conditions [13]. They associate this strategy with ppGpp-mediated control of the synthesis of ribosomal proteins [30–32]. The signalling molecule ppGpp accumulates in response to an increase in the level of uncharged tRNA, when amino acid concentrations in the cell drop. This causes ribosomes to “stall” and leads to RelA-mediated conversion of GTP to ppGpp, the molecular details of which are still subject of debate [32, 45]. Since ppGpp inhibits the transcription of ribosomal RNAs [46], the concentration of the latter decreases, leading to more inactive ribosomal proteins and, through a well-characterized post-transcriptional autoregulatory mechanism, a lower synthesis rate of ribosomal proteins [31, 47]. Our analysis adds to the above study a novel insight: measuring precursors does not only enable resource allocation control to achieve maximal growth at steady state, but is also a good strategy in a dynamical context.

While the precursor-only strategy is thus seen to lead to good results, Fig 6*D* shows that there remains room for improvement. It seems reasonable to expect that control strategies exploiting information of not just a single variable, but several variables simultaneously, could further improve the performance of the self-replicator during a growth transition.

### A near-optimal feedback control strategy: exploiting information on the imbalance between precursors and the gene expression machinery

In the quest for further improvements, a natural starting-point would be to consider the curve defining the optimal steady states $\left({\hat{p}}_{opt}^{*},{\hat{r}}_{opt}^{*}\right)$ for different environments *E*_*M*_. This curve is defined by a function mapping ${\hat{p}}^{*}$ to ${\hat{r}}^{*}$, which is actually the same as the function *g* introduced in the precursor-only strategy (Methods and S1 Fig), given that at steady state $\hat{r}=\alpha$ (Eq 12). The curve can be seen as representing an optimal balance between precursors and the gene expression machinery, in the sense that the maximal growth rate attainable for a given precursor concentration $\hat{p}$ requires a concentration $\hat{r}$ of ribosomes and other components of the gene expression machinery equal to $g(\hat{p})$. If either $\hat{r}>g\left(\hat{p}\right)$ or $\hat{r}<g\left(\hat{p}\right)$, the growth rate is suboptimal.

These considerations suggest an intuitive control strategy, namely to avoid an imbalance between $\hat{p}$ and $\hat{r}$ at all times, and remain as close as possible to the curve defined by *g*. In particular, when the gene expression machinery is more abundant than what is optimal given the available precursors ($\hat{r}>g\left(\hat{p}\right)$), its synthesis is switched off (*α* = 0). Conversely, when $\hat{r}<g\left(\hat{p}\right)$, synthesis of the gene expression machinery is switched on. This strategy thus tries to restore “as quickly as possible” the optimal balance between precursors $\hat{p}$ and the gene expression machinery $\hat{r}$, giving rise to a so-called on-off control strategy:

$$\alpha =h\left(\hat{p},\hat{r}\right)=\left\{\begin{array}{c} 0,\;\text{if}\;\hat{r}>g\left(\hat{p}\right),\\ 1,\;\text{if}\;\hat{r}<g\left(\hat{p}\right),\\ \alpha_{opt}^{*}\;\text{if}\;\left(\hat{p},\hat{r}\right)=\left({\hat{p}}_{opt}^{*},{\hat{r}}_{opt}^{*}\right). \end{array}\right.$$ (19)

As shown in the Methods section, the on-off strategy drives the system to a stable steady state ensuring growth at the maximal rate. Notice that, contrary to the strategies discussed in the previous section, the value of *α* selected by the on-off strategy depends on both $\hat{p}$ and $\hat{r}$ (Fig 5). It thus uses more information on the state of the system than the nutrient-only and precursor-only strategies.

**Fig 7:**
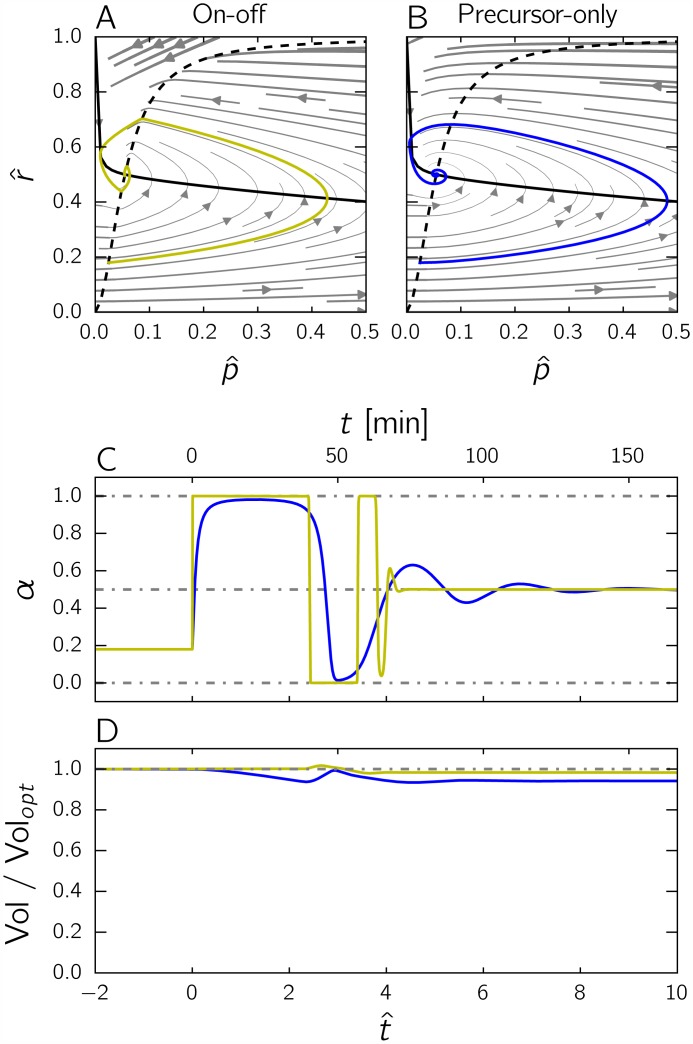
Comparison of the performance of the precursor-only and the on-off strategies after a nutrient upshift. *A:* Trajectory in the phase plane for the on-off strategy (yellow curve). The solid, black curve represents the $\hat{p}$-nullcline and the dashed, black curve the function *g*. The solution is obtained by numerical simulation of the system of Eqs 11 and 12, supplemented with the equation $\alpha =h(\hat{p},\hat{r})$ defined in Eq 19 and plotted in Fig 8*A*. The initial state corresponds to the optimal steady state attained for an environment given by 0.2*E*_*M*_. *B:* Trajectory in the phase plane for the precursor-only strategy (same as in Fig 6*B*, added for comparison). *C:* Evolution of the control variable *α* for each strategy as a function of time. Both strategies stabilize the system at the optimal steady state, but only the on-off strategy (yellow curve) exhibits bang-bang behavior. *D:* Evolution of the ratio Vol/Vol_*opt*_ for the on-off and precursor-only strategies as a function of time, where Vol is the volume of the self-replicator and Vol_*opt*_ the volume of the same replicator following the optimal strategy shown in Fig 4. The final values of Vol/Vol_*opt*_ attained by the two strategies are 0.9831 and 0.9413, respectively. The on-off strategy is thus hardly distinguishable from the optimal control strategy in the plot. In all of the above simulations, the parameter values *E*_*M*_ = 1 and *K* = 0.003 were used.

Fig 7 shows the performance of the on-off strategy after a nutrient upshift, as compared to the precursor-only strategy. The transition is seen to be nearly perfect, in the sense that 98% of the optimal biomass is produced by the strategy. The time course of *α* in panel *D* is very similar to the optimal time course obtained by numerical optimization, shown in Fig 4*B*, and clearly brings out the bang-bang-singular nature of the solution. These results show that a strategy exploiting complete information on the internal state of the self-replicator can lead to near-optimal performance, outcompeting a strategy that uses partial information on the internal state (precursor abundance only).

Are microbial cells equipped with mechanisms implementing a strategy similar to the on-off strategy? A possible candidate would again be the ppGpp system. A kinetic model of ppGpp metabolism and the regulation of the synthesis of ribosomal proteins was recently presented by Bosdriesz *et al*. [17]. The model proved capable of accounting for a range of experimental data, including the steady-state concentration of ppGpp as a function of the growth rate [48] and the dynamical response of ppGpp to a nutrient upshift or downshift [49]. A major conclusion of the model is that the steady-state concentration of ppGpp exhibits a strongly ultrasensitive response to deviations of the ribosomal protein fraction from the optimal ribosomal protein fraction at a given growth rate. These deviations from optimality, in turn, lead to a switch-like response of the synthesis rate of ribosomal proteins (Fig. 4 in Bosdriesz *et al*. [17]).

How does this mechanistic model of ppGpp regulation relate to the on-off strategy presented above? In order to answer this question, we first need to find a correspondence between the variables *p* and *r* of our coarse-grained model and the concentrations of molecular species in the kinetic model of Bosdriesz *et al*. This is rather straightforward to achieve, by equating *p* to the total amino acid concentration and *r* to the ribosome concentration. Second, S4 Text shows that by making two simplifying assumptions, ppGpp can be expressed as a function of the total amino acid concentration and the ribosome concentration. In particular, we assume that concentrations of all individual amino acids are equal, and that the concentrations of charged tRNAs and ppGpp evolve fast relative to the dynamics of the amino acid and ribosome concentrations. The third step consists in positing an explicit relation between ppGpp and *α*, based on the regulatory action of ppGpp on the transcription of ribosomal RNA [46]:

$$\alpha \left(\cdot \right)=\frac{K_{I}}{K_{I}+\text{ppGpp}\left(\cdot \right)},$$ (20)

with *K*_*I*_ a Michaelis-Menten inhibition constant [*μ*mol L^-1^] and ppGpp the (time-varying) intracellular concentration of ppGpp [*μ*mol L^-1^].

**Fig 8:**
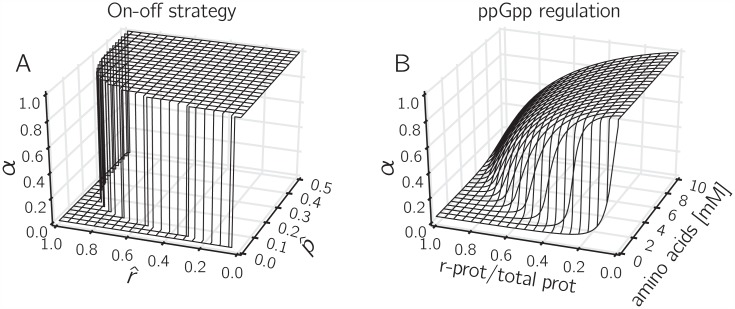
ppGpp regulation implements an on-off control strategy of resource allocation. *A:* Response surface of the on-off control strategy, defined by $\alpha =h(\hat{p},\hat{r})$ in Eq 19. *B:* Response surface of the ppGpp control strategy, as defined by Eq 20 and the simplified kinetic model defining ppGpp in terms of the total amino acid concentration and the ribosomal protein fraction (S4 Text). The shape of the response surface of the ppGpp control strategy is seen to be in very good agreement with the on-off strategy leading to near-optimal performance of the self-replicator during a nutrient upshift.

The response function for ppGpp thus obtained and evaluated for a range of amino acid and ribosome concentrations is represented in Fig 8, and visually compared with the on-off strategy. As can be seen, the two response surfaces are very similar. In other words, the ultrasensitive response of the synthesis rate of ribosomal proteins to the suboptimal allocation of cellular resources, derived from a model of the molecular mechanisms involved in the synthesis, degradation, and regulatory action of ppGpp [17], implements a control strategy that is close to the optimal predicted by a control-theoretical analysis of the self-replicator. While the role of ppGpp in maintaining optimal resource allocation was already pointed out by Scott *et al*. and Bosdriesz *et al*., the latter studies were restricted to optimizing steady-state growth. A major insight from the analysis in this section is that this conclusion seems to carry over to dynamical scenarios as well. Fundamentally, the analysis suggests that the ppGpp system is a likely candidate to fulfill this role because it integrates information on the imbalance between precursor concentration and abundance of the gene expression machinery.

## Discussion

Quantitative growth laws are empirical regularities pointing at fundamental properties of microbial life [50]. Recent work has led to the precise theoretical formulation of growth laws and has shown that they can be derived from basic assumptions on the molecular processes responsible for the assimilation of nutrients and their conversion to biomass [11, 13, 15, 17, 18]. The growth laws are uniquely defined under the hypothesis that microorganisms allocate resources in such a way as to maximize their growth rate. Several of the above-mentioned studies have analyzed feedback control strategies on the molecular level enabling cells to achieve optimal resource allocation in a robust manner. The control strategies exploit information on the physiological state of the cell to adjust the (relative) rate of synthesis of different classes of proteins (ribsomes, metabolic enzymes, …). Whereas the growth laws describe microbial growth at steady state, most microorganisms live in complex, continuously changing environments. Despite some precursory work [25, 26], questions about the dynamics of microbial growth remain largely unanswered: Which resource allocation schemes are optimal in changing environments? Which dynamical control strategies lead to (near-)optimal resource allocation? How do these strategies compare with those actually implemented by microorganisms?

We have addressed the above questions by means of a self-replicator model of microbial growth, which, like other coarse-grained models of bacterial growth [11, 13, 15], is capable of reproducing the growth laws at steady state (Fig 3). A first major contribution of our work is to show that, in the case of a dynamical upshift scenario, optimal production of biomass requires a bang-bang-singular resource allocation scheme (Fig 4). That is, the optimal self-replicator should iteratively allocate all of its resources to the gene expression machinery (bang control input) and the metabolic machinery (another bang control input), until the steady state enabling maximal growth in the post-upshift environment is reached, corresponding to a trade-off in the allocation of resources to the two processes (singular control input).

Bang-bang phenomena are widespread in a variety of life processes. Applications of optimal control theory to reproductive strategies in insects [51], the development of intestinal crypts [52], and the activation of metabolic pathways [53, 54] have led to bang-bang or bang-bang-singular strategies. In optimal control problems, such a solution arises with systems where the differential equations are linear in the control variable (in our case, *α*(⋅)). Examples of applications that are close to the problem considered here are the control of gene expression for adaptation to environmental changes [25, 55], and the allocation of resources between nutrient uptake and growth in microorganisms [26, 56]. Whereas the former applications focus on minimization of response times, the latter also optimize biomass during a growth transition, using a different model, not derived from first principles as in this study. However, the optimal solution of the corresponding optimal control problem is also bang-bang-singular, thus showing that our conclusions are robust to model variations.

Our second major contribution is the assessment of how different feedback control strategies perform with respect to each other and to the gold standard determined from optimal control theory. We show that the precursor-only and nutrient-only strategies, both of which drive the self-replicator to the steady state with maximal growth rate in a static environment, perform quite differently in a dynamical upshift scenario (Fig 6). While the precursor-only strategy is better than the nutrient-only strategy in a dynamical environment, it is in turn outperformed by a so-called on-off strategy, which achieves a near-perfect growth transition by exploiting information on the imbalance between the precursor concentration and the abundance of the gene expression machinery (Fig 7). The superior performance of the on-off strategy can be intuitively explained by the fact that during a growth transition the two variables are not fully correlated, which means that sensing both instead of either one provides additional information in a dynamical context.

Interestingly, the on-off strategy is based on a feedback control law that very much resembles the response function for ppGpp-mediated regulation of the synthesis of ribosomal RNAs in *E. coli* [17]. The role of ppGpp in controlling microbial growth has been amply documented [30–32]. For example, Potrykus *et al*. observed that in cells without ppGpp (ppGpp^0^ mutants) the RNA/protein mass ratio, a proxy for our resource allocation variable *α*, does not change with the growth rate, which has led these authors to conclude that ppGpp is the major source of growth-rate control in *E. coli* [57]. The central importance of ppGpp in the reallocation of gene expression resources in *E. coli* following changes in nutrient availability has also been mapped with higher resolution, using genome-wide transcriptome studies [58, 59]. In nearly all bacterial species examined so far, ppGpp is known to accumulate in response to an increase in the level of uncharged tRNA [60], although the molecular details of ppGpp metabolism and the range of other functions of the alarmone may greatly vary across species [32, 60, 61]. While it has thus been well-established that regulation by ppGpp is an evolutionary conserved mechanism of growth control in the bacterial cell, our analysis provides a new perspective by suggesting that ppGpp enables optimal reallocation of resources after a growth transition, dynamically maximizing the accumulation of biomass.

The model on which the above results are based is built from first principles by distinguishing two fundamental cellular processes: metabolism (converting nutrients to precursors) and gene expression (converting precursors to the proteins that make up biomass) (Fig 1). Despite its simplicity, our self-replicator model is capable of reproducing the empirical growth laws and of making testable predictions on the time-course profile of the resource allocation variable *α* and on the concentrations *p* and *r* of components of the gene expression machinery and metabolic machinery, respectively (see Fig 8 and below). The model can be easily extended with more details on protein synthesis, central carbon and energy metabolism, stress systems, or cell membranes, but this would make the mathematical analysis of the model dynamics and the optimal control problem more complicated. Notice, however, that the direct numerical approach for solving the optimal control problem remains applicable, even for more fine-grained models (Fig 4, see also [27]).

The comparison of different control strategies during a classical growth transition should be interpreted carefully, in a qualitative rather than quantitative manner. Whereas the differences in performance based on the biomass ratio Vol/Vol_*opt*_ of the control strategies are robust, the absolute numbers for the biomass ratio will depend on details of the growth experiment chosen and the exact parameter values. Another implicit assumption in the analysis of the control strategies is that the costs of their molecular implementation can be neglected. This is not true in general, since every control strategy requires resources to be diverted towards the synthesis of sensory systems and regulatory proteins, with possibly detrimental effects on growth. In other words, a control strategy entails a trade-off between the growth burden of regulation and the growth benefit of the improved capability to adapt to changes in the environment [62, 63]. The analysis of control strategies could be refined by adding a reaction to the self-replicator that models the loss of resources incurred by regulatory strategies. While in general the growth burden of a control strategy requiring information on several aspects of cellular physiology is expected to be higher, notice that a single regulatory system may be capable of sensing more than one variable. For example, we show that ppGpp levels in the cell carry information on both the metabolic and the gene expression state (Fig 8), thus integrating several signals in a cost-efficient manner.

The model predictions for the dynamical adaptation of resource allocation after a nutrient upshift suggest several interesting experimental tests. In particular, the switching profile of the resource allocation variable *α* is a promising candidate for experimental validation. The most straightforward option would be direct measurement of the synthesis rate of ribosomal proteins, using a translational fusion of a fluorescent reporter with a ribosomal protein [45, 64]. However, a more indirect approach based on the quantification of ppGpp concentrations in the cell or the activity of the ribosomal RNA (rRNA) promoters would also be a possibility. Interestingly, some data are already available in the literature. For instance, Gausing has reviewed data on the synthesis of ribosomal proteins after a nutrient upshift, showing that the synthesis rate goes through “a series of rapid changes” resembling oscillations [65]. Later work attributed this pattern to regulation on the transcriptional level [66]. Friesen *et al*. observed oscillatory patterns in ppGpp concentrations after a nutrient upshift, with an initial response resembling bang control for an upshift to a particularly rich medium [67]. Murray *et al*. also present data on the ppGpp concentration after a nutrient upshift [49], but with a lower temporal resolution and no clear oscillatory pattern. All of the above measurements were carried out on the population level, which means that switching patterns may be obscured by desynchronisation of the individual cells. More sophisticated experimental set-ups are necessary for the decisive validation of the model predictions, allowing gene expression in single cells to be followed over time in tightly regulated growth conditions [68, 69]. In addition, the model could be validated on other dynamical scenarios, for example nutrient downshifts [49, 70].

Apart from its interest for fundamental science, resource allocation is also a critical question in biotechnology, where there exists an inherent trade-off between the maximization of yield and productivity [71]. High yield means that most of the substrate is converted to a metabolite, peptide or recombinant protein of interest, but this leads to low productivity if the remaining nutrient influx is insufficient to sustain population growth. Engineered control of resource allocation may help in establishing the right trade-off, the most profitable balance between yield and productivity, in a biotechnological process. Such a trade-off could be attained either in steady-state conditions (the incoming nutrient flux is optimally distributed over growth and production) or in dynamical conditions (alternating utilization of the incoming nutrient flux for growth or production) [72–74]. When extended with heterologous metabolic pathways, the self-replicator models used in this study would provide an adequate *in-silico* test bed for the rapid screening and comparison of alternative control strategies in bioprocess engineering.

## Methods

### Steady-state analysis of model

The nondimensional version of the model, given by Eqs 11 and 12, was used for a steady-state analysis of the self-replicator. Eqs 11 and 12 were derived from the original model of Eqs 3 and 4 by means of the following rescalings:

$$\hat{p}=\beta \;p,\;\;\;\hat{r}=\beta \;r,\;\;\;\hat{t}=k_{R}\;t,\;\;\;E_{M}=e_{M}/k_{R},\;\;\;K=\beta K_{R}.$$

As shown in S1 Text, for a constant environment *E*_*M*_ and constant resource allocation *α*, the system has two steady states: a trivial unstable steady state $\left({\hat{p}}^{*},{\hat{r}}^{*}\right)=\left(0,1\right)$, allowing no growth in the absence of precursors, and a steady state with a positive growth rate given by

$$\left({\hat{p}}^{*},{\hat{r}}^{*}\right)=\left(\frac{\left(1-\alpha \right)\;E_{M}-\alpha +\sqrt{{\left[\left(1-\alpha \right)\;E_{M}-\alpha \right]}^{2}+4\alpha \;\left(1-\alpha \right)\;E_{M}\;K}}{2\alpha },\alpha \right).$$ (21)

The two eigenvalues of the Jacobian matrix evaluated at $\left({\hat{p}}^{*},{\hat{r}}^{*}\right)$ are negative (S1 Text), so that this steady state is stable.

The growth rate at steady state, as a function of ${\hat{p}}^{*}$ and ${\hat{r}}^{*}$, is given by Eq 13, which we repeat here for clarity:

$${\hat{\mu }}^{*}=\frac{{\hat{p}}^{*}}{K+{\hat{p}}^{*}}\;{\hat{r}}^{*}.$$

Evaluating $d\hat{p}/dt=0$ at $\left({\hat{p}}^{*},{\hat{r}}^{*}\right)$ allows ${\hat{r}}^{*}$, and therefore ${\hat{\mu }}^{*}$, to be written as a function of ${\hat{p}}^{*}$ (S1 Text). Accordingly, we can compute $\partial {\hat{\mu }}^{*}/\partial {\hat{p}}^{*}$ and, when setting this partial derivative to 0, determine the maximum growth rate at steady state $\mu_{opt}^{*}$ and the optimal resource allocation $\alpha_{opt}^{*}$ bringing about this maximal growth rate. As shown in S1 Text, $\mu_{opt}^{*}$ and $\alpha_{opt}^{*}$ can be written as explicit functions of either the environment *E*_*M*_:

$$\alpha_{opt}^{*}=\frac{E_{M}+\sqrt{K\;E_{M}}}{E_{M}+2\sqrt{K\;E_{M}}+1},\;\;\;\;\;\;\;\;\;\;\;\;\;\;{\hat{\mu }}_{opt}^{*}=\frac{E_{M}}{E_{M}+2\sqrt{K\;E_{M}}+1},$$ (22)

or the precursor abundance ${\hat{p}}_{opt}^{*}$:

$$\alpha_{opt}^{*}=\frac{{\hat{p}}_{opt}^{*}}{{\hat{p}}_{opt}^{*}+\frac{K}{K+{\hat{p}}_{opt}^{*}}\left(1+{\hat{p}}_{opt}^{*}\right)},\;\;\;\;\;\;\;\;\;\;\;\;\;\;{\hat{\mu }}_{opt}^{*}=\frac{{\hat{p}}_{opt}^{*2}}{{\hat{p}}_{opt}^{*2}+2K{\hat{p}}_{opt}^{*}+K}.$$ (23)

The above equations were used for the derivation of the control strategies (see below).

### Model fitting

As can be seen by comparing Fig 3*A* and 3*B*, growth-rate maximization in the self-replicator model leads to a good qualitative correspondence with the growth laws. In order to determine if a good quantitative fit of the model with the data from Scott *et al*. [12] can be obtained, for reasonable parameter values, we estimated *e*_*M*_ and *k*_*R*_ in Eqs 3 and 4 from the measured RNA/protein mass ratios. At steady state, the RNA/protein mass ratio can be interpreted as proportional to ${\hat{r}}^{*}$ (and thus $\alpha_{opt}^{*}$), with an unknown (dimensionless) proportionality constant *γ* (see [12] for details on the use of the RNA/protein mass ratio as a proxy for the ribosomal protein mass fraction):

$${\hat{r}}^{*}=\alpha_{opt}^{*}=\gamma \;\frac{\text{RNA}\;\text{mass}}{\text{protein}\;\text{mass}}.$$ (24)

Reformulating Eq 22 in terms of the original parameters *e*_*M*_ and *k*_*R*_, which have physical dimensions facilitating the biological interpretation of their values, we obtain a straighforward relation between *e*_*M*_, *k*_*R*_, *K*, $\alpha_{opt}^{*}$ and $\mu_{opt}^{*}$:

$$\alpha_{opt}^{*}=\frac{e_{M}+\sqrt{K\;e_{M}\;k_{R}}}{e_{M}+2\sqrt{K\;e_{M}\;k_{R}}+k_{R}},\;\;\;\;\;\;\;\;\;\;\;\;\;\;\mu_{opt}^{*}=\frac{e_{M}\;k_{R}}{e_{M}+2\sqrt{K\;e_{M}\;k_{R}}+k_{R}}.$$ (25)

Eqs 24 and 25 were used to estimate values of *k*_*R*_ and *γ*, as well as *e*_*M*_ for each of the six growth conditions, from the measurements of the growth rate and the RNA/protein mass ratio. The value *K* was not estimated from the experimental data, but set to a value inferred from the literature (S1 Text). The optimization process was carried out by means of the differential evolution algorithm of Storn and Price [75]. The results are shown in Fig 3*B*, while the estimated parameter values are summarized in S1 Table. The parameter values are in very good agreement with order-of-magnitude values determined from the literature (S2 Text and S1 Table).

### Solution of optimal control problem

The optimal control problem of Eq 10 consists in identifying the function *α*_*opt*_(*t*) that maximizes the integral of the growth rate $\hat{\mu }$ over an interval [0, *τ*]. In order to solve this problem, we first redefined it over an infinite horizon (*i.e*., *τ* → ∞) in order to avoid boundary effects occurring over finite time intervals, in particular the depletion of precursors just before reaching *τ*. With $U=\{\alpha :R^{+}\to \left[0,1\right]\}$ the set of admissible controls, the full optimization problem for the nondimensionalized system is given by

$$\max_{\alpha \in U}J\left(\alpha \right)\equiv \int_{0}^{\infty }\hat{r}\left(\hat{t}\right)\;\frac{\hat{p}\left(\hat{t}\right)}{K+\hat{p}\left(\hat{t}\right)}\;d\hat{t}.$$ (26)

Since *J*(*α*) diverges, we actually consider overtaking optimality: A solution is overtaking optimal if its performance index catches up with the performance index of any other solution ([40], see S3 Text for details).

Necessary conditions on optimal trajectories can be obtained by the Infinite Horizon Maximum Principle [40], an extension of the well-known Pontryagin Maximum Principle. Analysis of the Hamiltonian of the system of Eqs 11 and 12 and the associated adjoint system shows that the optimal trajectory is a concatenation of bang arcs (*α*(⋅) = 0 or *α*(⋅) = 1) and possibly a singular arc corresponding to the optimal steady state $\left(\hat{p}\left(t\right),\hat{r}\left(t\right)\right)=\left({\hat{p}}_{opt}^{*},{\hat{r}}_{opt}^{*}\right)$, that is, the steady state leading to the optimal growth rate ${\hat{\mu }}_{opt}^{*}$ in the new environment after the upshift (S3 Text). Moreover, from the Kelley condition [76], we can show that if the optimal trajectory has a singular arc, then it must enter this singular arc *via* a chattering arc, *i.e*., with an infinite number of switches of *α*(⋅) between 0 and 1 (S3 Text). The chattering arc is characterized by a switching curve $\hat{r}=\varphi \left(\hat{p}\right)$ in the $\left(\hat{p},\hat{r}\right)$-plane, which passes through $\left({\hat{p}}_{opt}^{*},{\hat{r}}_{opt}^{*}\right)$. The switching curve divides the phase plane into two regions, such that *α*(*t*) switches to 0 when the system is in the region above *φ* and to 1 when the system is below *φ* (S3 Text and Fig 4).

The above results have led to the conjectured optimal solution of Eq 14. In parallel, we numerically solved the problem of Eq 26 by a direct method using the bocop software [41]. A time discretization allows the optimal control problem to be transformed into a nonlinear optimization problem, solved here by interior point techniques. A discretization by a Lobatto IIIC formula (6th order) was used with 4000 time steps, and the relative tolerance for the NLP solver was set to 10^−14^. The optimal trajectories thus obtained are composed of a chattering arc followed by a steady state corresponding to the singular arc (Fig 4). The switching curve $\varphi (\hat{p})$ was computed from numerical simulations with different initial conditions.

### Specification and analysis of control strategies

As described in the *Results* section, we are interested in control strategies satisfying the following conditions:

(C1) The control laws are static functions of the system variables (as opposed to, for instance, functions that depend on derivatives or integrals of the variables).
(C2) For any given constant environment *E*_*M*_, they drive the self-replicator system towards a unique stable steady state that is not trivial, *i.e*., with nonzero growth rate.
(C3) This steady state corresponds to the optimal steady state $\left({\hat{p}}_{opt}^{*},{\hat{r}}_{opt}^{*}\right)$, allowing growth at the maximal rate $\mu_{opt}^{*}$.

It can be directly verified from the functions *f*, *g*, and *h* defining the nutrient-only, precursor-only, and on-off control strategies (Eqs 15, 17 and 19) that they are indeed static functions of the system variables (or the system input, in the case of the nutrient-only strategy). Here we show that the other two conditions are also satisfied for all three strategies.

Following Eq 15, the nutrient-only strategy is defined by *α* = *f*(*E*_*M*_), so that *α* is constant after the upshift. As shown above and in S1 Text, this means that the system controlled by the nutrient-only strategy has a single nontrivial stable steady state (Condition C2). In addition, in this case the optimal steady state is attained for $\alpha_{opt}^{*}$ defined as in Eq 22, and the following function *f* therefore guarantees Condition C3:

$$f\left(E_{M}\right)=\frac{E_{M}+\sqrt{KE_{M}}}{E_{M}+2\sqrt{K\;E_{M}}+1}.$$ (27)

In S1 Text, it is shown that Eq 27 is the only definition of *f* satisfying all conditions. S1 Fig shows a plot of *f*(*E*_*M*_) together with a biologically plausible Michaelis-Menten approximation (Eq 16).

The full specification of the precursor-only strategy demands an expression for the function *g* in Eq 17. Recall that Eq 23 defines $\alpha_{opt}^{*}$ in terms of the precursor concentration ${\hat{p}}_{opt}^{*}$, which leads us to propose the following function *g*:

$$g\left(\hat{p}\right)=\frac{\hat{p}}{\hat{p}+\frac{K}{K+\hat{p}}\left(1+\hat{p}\right)}.$$ (28)

As shown in S1 Text by computing the Jacobian, the system given by Eqs 11, 12 and 28 has a single nontrivial stable steady state for any environment *E*_*M*_ (Condition C2). Moreover, Eq 28 guarantees this steady state to be optimal (Condition C3). This can be seen by noting that at steady state, $d\hat{r}/dt=0$ implies ${\hat{r}}^{*}=g\left({\hat{p}}^{*}\right)$ (Eq 12). In order for the self-replicator to attain a maximal growth rate at steady rate, Eq 23 needs to be satified, which is the case for the above choice of the function *g*. Like for *f*, Eq 28 is the only choice for *g* satisfying C1–C3. S1 Fig shows a plot of $g(\hat{p})$ together with a biologically plausible Hill approximation (Eq 18).

The on-off control strategy is defined in Eq 19 and repeated below:

$$h\left(\hat{p},\hat{r}\right)=\left\{\begin{array}{c} 0,\;\text{if}\;\hat{r}>g\left(\hat{p}\right),\\ 1,\;\text{if}\;\hat{r}<g\left(\hat{p}\right),\\ \alpha_{opt}^{*},\;\text{if}\;\left(\hat{p},\hat{r}\right)=\left({\hat{p}}_{opt}^{*},{\hat{r}}_{opt}^{*}\right). \end{array}\right.$$ (29)

This strategy drives the system to a single steady state, because the $\hat{p}$-nullcline crosses the function $g(\hat{p})$ only once, as shown graphically in Fig 7*A*. In S1 Text we argue that this steady state is stable, by taking into account so-called sliding modes on the switching curve [77] (Condition C2). Moreover, the steady state coincides with the optimal steady state $\left({\hat{p}}_{opt}^{*},{\hat{r}}_{opt}^{*}\right)$ by construction, so that Condition C3 is satisfied as well. Fig 8*A* shows a plot of $h(\hat{p},\hat{r})$.

Note that since *h*(⋅) is discontinuous, numerical instabilities occur during simulations. We therefore used the following continuous approximation of this function:

$$\frac{g{\left(\hat{p}\right)}^{100}}{g{\left(\hat{p}\right)}^{100}+{\hat{r}}^{100}},\;\text{if}\;\hat{r}\neq g\left(\hat{p}\right).$$ (30)

The approximation causes *α* to take intermediate values (instead of 0 or 1) just before reaching the optimal steady state in Fig 7*C*. For numerical simulations of the ODE system, we used the CVODE solver [78] from SUNDIALS 2.6.2 [79].

## Supporting Information

S1 Text Model derivation and analysis. (PDF)

S2 Text Model parameters. (PDF)

S3 Text Solution of optimal control problem. (PDF)

S4 Text Kinetic model of the ppGpp system in *Escherichia coli*. (PDF)

S1 Table Parameter values of self-replicator model. (PDF)

S1 Fig Simple control strategies for the self-replicator of bacterial growth. (PDF)
