# Supplementary material for: Dynamical Allocation of Cellular Resources as an Optimal Control Problem: Novel Insights into Microbial Growth Strategies
Source: PLoS Comput Biol. 2016 Mar 9;12(3):e1004802. doi: 10.1371/journal.pcbi.1004802 (PMC4784908; doi:10.1371/journal.pcbi.1004802)
Supplement: S1 Text — (PDF) [file pcbi.1004802.s001.pdf]

S1 Text – Model derivation and analysis[1]

Nils Giordano [1,3], Francis Mairet [2], Jean-Luc Gouzé [2], Johannes Geiselmann [1,3,*], Hidde de Jong [3,*]

1 Université Grenoble Alpes, Laboratoire Interdisciplinaire de Physique (CNRS UMR 5588), 140 rue de la Physique BP 87, 38402 Saint Martin d'Hères, France
2 Inria, Sophia-Antipolis Méditerranée research centre, 2004 route des Lucioles, BP 93, 06902 Sophia-Antipolis Cedex, France
3 Inria, Grenoble - Rhône-Alpes research centre, 655 avenue de l'Europe, Montbonnot, 38334 Saint Ismier Cedex, France

*** Corresponding authors with equal contributions:**
Hidde.de-Jong@inria.fr, Hans.Geiselmann@ujf-grenoble.fr

S1.1 Model formulation

The time evolution of the total mass of each component of the self-replicator can be written as follows:

$$\begin{aligned} \frac{dP}{dt} &= V_M(t) - V_R(t), \\ \frac{dM}{dt} &= (1-\alpha(t))\, V_R(t), \\ \frac{dR}{dt} &= \alpha(t)\, V_R(t), \end{aligned} \tag{S1.1}$$

where P, M, R [g] denote the total mass of precursors, metabolic machinery and gene expression machinery, respectively. V_M [g h^{-1}] is the rate of production of precursors by metabolism and V_R [g h^{-1}] the rate of utilisation of precursors for gene expression.

Dividing the mass variables by the total time-varying volume $\texttt{Vol}(t)$ of the system, we obtain the concentration variables $p = P/\texttt{Vol}$, $m = M/\texttt{Vol}$, $r = R/\texttt{Vol}$ [g L^{-1}]. The dynamics of the concentration variables then follows with Eq. S1.1:

$$\begin{aligned} \frac{dp}{dt} &= \frac{V_M(t)}{\texttt{Vol}} - \frac{V_R(t)}{\texttt{Vol}} - \frac{1}{\texttt{Vol}}\frac{d\texttt{Vol}}{dt}\,p, \\ \frac{dm}{dt} &= (1-\alpha(t))\frac{V_R(t)}{\texttt{Vol}} - \frac{1}{\texttt{Vol}}\frac{d\texttt{Vol}}{dt}\,m, \\ \frac{dr}{dt} &= \alpha(t)\,\frac{V_R(t)}{\texttt{Vol}} - \frac{1}{\texttt{Vol}}\frac{d\texttt{Vol}}{dt}\,r. \end{aligned} \tag{S1.2}$$

At this point, we define $v_M = V_M/\texttt{Vol}$ and $v_R = V_R/\texttt{Vol}$ [g L^{-1} h^{-1}] as the mass fluxes per unit volume. Moreover, with the definition of the volume in terms of the total protein mass in Eq. 2 of the main text, that is, $\texttt{Vol} = \beta\,(M+R)$, we find that

$$\frac{1}{\texttt{Vol}}\frac{d\texttt{Vol}}{dt} = \frac{\beta}{\texttt{Vol}}\frac{d(M+R)}{dt} = \beta\,\frac{V_R(t)}{\texttt{Vol}} = \beta\, v_R(t). \tag{S1.3}$$

[1]Supporting Information of "Dynamical Allocation of Cellular Resources as an Optimal Control Problem: Novel Insights into Microbial Growth Strategies "

This leads to the system

$$\frac{dp}{dt} = v_M(t) - v_R(t)\,(1 + \beta\, p), \tag{S1.4}$$

$$\frac{dr}{dt} = v_R(t)\,(\alpha(t) - \beta\, r), \tag{S1.5}$$

where the equation for $m(t)$ is omitted since by construction $r(t) + m(t) = 1/\beta$ and $dr/dt + dm/dt = 0$.

As stated in the main text, we use Michaelis-Menten kinetics to express v_M and v_R in terms of the system variables:

$$v_M(t) = m(t)\,k_M\,\frac{s(t)}{K_M + s(t)} = \left(\frac{1}{\beta} - r(t)\right) e_M(t),$$

$$v_R(t) = r(t)\,k_R\,\frac{p(t)}{K_R + p(t)},$$

with rate constants k_M, k_R [h^{-1}] and half-saturation constants K_M, K_R [$\mathrm{g\ L}^{-1}$]. $s(t)$ is an exogenous variable representing the nutrient concentration in the external medium. We simplify $v_M(t)$ by defining the environmental input $e_M(t) = k_M\,s(t)/(K_M + s(t))$. Throughout the paper, as explained in the main text, we assume the environment is constant, *i.e.*, $e_M(t) = e_M$.

Finally, the growth rate μ [h^{-1}] is defined as the relative increase of the volume of the self-replicator. From Eq. S1.3, it follows that:

$$\mu(t) = \frac{1}{\mathtt{Vol}}\frac{d\mathtt{Vol}}{dt} = \beta\, v_R(t). \tag{S1.6}$$

S1.2 Nondimensionalization of the system

For the sake of simplifying the proofs and derivations below, we define the following nondimensional variables:

$$\hat{p} = \beta\, p, \quad \hat{r} = \beta\, r, \quad \hat{t} = k_R\, t.$$

When injecting these into Eq. S1.4, we obtain

$$\frac{k_R}{\beta}\frac{d\hat{p}}{d\hat{t}} = \left(\frac{1}{\beta} - \frac{\hat{r}}{\beta}\right) e_M - \frac{\hat{r}}{\beta}\,k_R\,\frac{\hat{p}}{\beta K_R + \hat{p}}\,(1 + \hat{p}),$$

which simplifies to

$$\frac{d\hat{p}}{d\hat{t}} = (1 - \hat{r})\,\frac{e_M}{k_R} - \hat{r}\,\frac{\hat{p}}{\beta K_R + \hat{p}}\,(1 + \hat{p}).$$

In a similar manner, we derive the time evolution of the nondimensional $\hat{r}$, and thus obtain the system

$$\begin{aligned} \frac{d\hat{p}}{d\hat{t}} &= (1 - \hat{r})\,E_M - (1 + \hat{p})\,\frac{\hat{p}}{K + \hat{p}}\,\hat{r}, \\ \frac{d\hat{r}}{d\hat{t}} &= (\alpha - \hat{r})\,\frac{\hat{p}}{K + \hat{p}}\,\hat{r}, \end{aligned} \tag{S1.7}$$

with the lumped parameters $E_M = e_M/k_R$ and $K = \beta\, K_R$. The corresponding nondimensionalized growth rate is given by

$$\hat{\mu} = \frac{\mu}{k_R} = \frac{\hat{p}}{K + \hat{p}}\,\hat{r}. \tag{S1.8}$$

S1.3 Steady-state growth of the self-replicator

If we suppose $E_M > 0$, $K > 0$ and $\alpha \in]0,1[$, there is a trivial unstable steady state at $(0,1)$. A second steady-state exists for the point in which $\hat{r}^* = \alpha$ and $\hat{p}^*$ is a root of the following polynomial:

$$\alpha\,\hat{p}^2 + (\alpha - (1-\alpha)\,E_M)\,\hat{p} - (1-\alpha)\,E_M\,K.$$

If we keep the only admissible root for this polynomial (*i.e.*, for which $\hat{p} \geq 0$), the second steady state is given by

$$(\hat{p}^*, \hat{r}^*) = \left(\frac{(1-\alpha)\,E_M - \alpha + \sqrt{[(1-\alpha)\,E_M - \alpha]^2 + 4\alpha\,(1-\alpha)\,E_M\,K}}{2\alpha}, \alpha \right). \tag{S1.9}$$

We can determine the stability of this steady state by looking at the Jacobian matrix J of the ODE system:

$$J = \begin{pmatrix} -\frac{\hat{r}}{K+\hat{p}} \left[\hat{p} + (1+\hat{p})\,\frac{K}{K+\hat{p}}\right] & -E_M - (1+\hat{p})\,\frac{\hat{p}}{K+\hat{p}} \\ (\alpha - \hat{r})\,\hat{r}\,\frac{K}{(K+\hat{p})^2} & (\alpha - 2\hat{r})\,\frac{\hat{p}}{K+\hat{p}} \end{pmatrix}. \tag{S1.10}$$

Evaluated at the point $(\hat{p}^*, \hat{r}^*)$, the Jacobian matrix becomes

$$J_{(\hat{p}^*,\hat{r}^*)} = \begin{pmatrix} -\frac{\alpha}{K+\hat{p}^*} \left[\hat{p}^* + (1+\hat{p}^*)\,\frac{K}{K+\hat{p}^*}\right] & -E_M - (1+\hat{p}^*)\,\frac{\hat{p}^*}{K+\hat{p}^*} \\ 0 & -\alpha\,\frac{\hat{p}^*}{K+\hat{p}^*} \end{pmatrix}.$$

Since $\hat{p}^*$, α, E_M, $K > 0$, the two eigenvalues are negative and therefore the steady state $(\hat{p}^*, \hat{r}^*)$ is stable (see also the streamlines in Figure 2*A* in the main text). It means that for fixed environmental conditions E_M and resource allocation α, the self-replicator converges towards a steady state in which the concentration variables are constant.

One can now easily derive the steady-state growth rate, denoted $\hat{\mu}^*$. By substituting Eq. S1.8 into the first ODE of the system of Eq. S1.7, we find at steady state:

$$\left(\frac{d\hat{p}}{d\hat{t}}\right)_{(\hat{p}^*,\hat{r}^*)} = 0 = (1-\alpha)\,E_M - (1+\hat{p}^*)\,\hat{\mu}^*,$$

which by means of Eq. S1.9 gives the following relation:

$$\hat{\mu}^* = \frac{(1-\alpha)\,E_M}{1+\hat{p}^*} = \frac{2\alpha(1-\alpha)\,E_M}{(1-\alpha)\,E_M + \alpha + \sqrt{[(1-\alpha)\,E_M - \alpha]^2 + 4\alpha(1-\alpha)\,E_M\,K}}. \tag{S1.11}$$

Finally, we can transform this expression to obtain

$$\hat{\mu}^* = \begin{cases} \frac{(1-\alpha)\,E_M + \alpha - \sqrt{[(1-\alpha)\,E_M - \alpha]^2 + 4(1-\alpha)\,\alpha\,E_M\,K}}{2(1-K)} & \text{for } K \neq 1, \\ \frac{\alpha\,(1-\alpha)\,E_M}{\alpha + (1-\alpha)\,E_M} & \text{for } K = 1. \end{cases} \tag{S1.12}$$

This function of α is plotted in Figure 2*B* in the main text.

S1.4 Maximization of growth rate at steady state

We are interested in the steady state at which growth occurs at the maximum rate. The growth rate at steady state $\hat{\mu}^*$ is given by

$$\hat{\mu}^* = \frac{\hat{p}^*}{K + \hat{p}^*}\,\hat{r}^*. \tag{S1.13}$$

From the first ODE of the system of Eq. S1.7, we have

$$\hat{r}^* = \frac{E_M}{E_M + \frac{\hat{p}^*}{K+\hat{p}^*}(1+\hat{p}^*)}. \tag{S1.14}$$

Substituting Eq. S1.14 into Eq. S1.13, we obtain

$$\hat{\mu}^* = \frac{E_M\,\hat{p}^*}{\hat{p}^{*2} + (E_M+1)\,\hat{p}^* + E_M\,K}. \tag{S1.15}$$

The value of $\hat{p}^*$ maximizing $\hat{\mu}^*$ can be determined from

$$\frac{\partial\hat{\mu}^*}{\partial\hat{p}^*} = \frac{E_M\,(E_M\,K - \hat{p}^{*2})}{\left(\hat{p}^{*2} + (E_M+1)\,\hat{p}^* + E_M\,K\right)^2}, \tag{S1.16}$$

by looking at the values of $\hat{p}^*$ for which this derivative equals 0. It follows that $\hat{\mu}^*$ is maximal for

$$\hat{p}^* = \hat{p}^*_{opt} = \sqrt{K\,E_M}. \tag{S1.17}$$

By substituting $\hat{p}^*_{opt}$ and α^*_{opt} for $\hat{p}^*$ and $\hat{r}^*$, respectively, in Eq. S1.14, we obtain the resource allocation maximizing the growth rate

$$\alpha^*_{opt} = \frac{E_M + \sqrt{KE_M}}{E_M + 2\sqrt{KE_M} + 1}. \tag{S1.18}$$

Finally, injecting this result into Eq. S1.13 we obtain the optimal steady-state growth rate:

$$\hat{\mu}^*_{opt} = \frac{E_M}{E_M + 2\sqrt{K\,E_M} + 1}. \tag{S1.19}$$

In addition, by using Eq. S1.17, we can write α^*_{opt} and $\hat{\mu}^*_{opt}$ as a function of $\hat{p}^*_{opt}$ only:

$$\alpha^*_{opt} = \frac{\hat{p}^*_{opt}}{\hat{p}^*_{opt} + \frac{K}{K+\hat{p}^*_{opt}}(1+\hat{p}^*_{opt})}, \qquad \hat{\mu}^*_{opt} = \frac{\hat{p}^{*2}_{opt}}{\hat{p}^{*2}_{opt} + 2K\hat{p}^*_{opt} + K}. \tag{S1.20}$$

S1.5 Analysis of the control strategies

In this section, we derive the main results for the functions f, g, and h defining the nutrient-only, precursor-only, and on-off control strategies. For each of these, we prove that the Conditions C1, C2 and C3 from the *Methods* section are satisfied, which we repeat here for clarity:

(C1) The control laws are static functions of the system variables (as opposed to, for instance, functions that depend on derivatives or integrals of the variables).

(C2) For any given constant environment E_M, they drive the self-replicator system towards a unique stable steady state that is not trivial, *i.e.*, with nonzero growth rate.

(C3) This steady state corresponds to the optimal steady state $(\hat{p}^*_{opt}, \hat{r}^*_{opt})$, allowing growth at the maximal rate μ^*_{opt}.

S1.5.1 Nutrient-only strategy

The nutrient-only strategy is defined by:

$$\alpha = f(E_M) = \frac{E_M + \sqrt{K E_M}}{E_M + 2\sqrt{K\, E_M} + 1}. \tag{S1.21}$$

It drives the system to the optimal steady state by measuring the environment E_M. Note that Condition C1 is satisfied by definition.

By injecting Eq. S1.21 into Eq. S1.7, the ODE system under the control of f becomes:

$$\begin{aligned} \frac{d\hat{p}}{d\hat{t}} &= (1-\hat{r})\, E_M - (1+\hat{p})\, \frac{\hat{p}}{K+\hat{p}}\, \hat{r}, \\ \frac{d\hat{r}}{d\hat{t}} &= (f(E_M) - \hat{r})\ \frac{\hat{p}}{K+\hat{p}}\, \hat{r}. \end{aligned} \tag{S1.22}$$

Since E_M is constant on the interval of interest (starting right after the upshift), we are in the case of Section S1.3 (*i.e.*, α constant). In particular, the system has two steady states: a trivial unstable one at (0, 1) (with zero growth), and a stable one defined by Eq. S1.9 (Condition C2). Since $f(E_M) = \alpha^*_{opt}$, we conclude from the derivations in Section S1.4 that the stable steady state is optimal for every environment E_M (Condition C3).

It is interesting to note that the expression in Eq. S1.21 is the only function $f(E_M)$ satisfying C1-C3. We can prove this statement by contradiction. Assume a control strategy $c(E_M)$ satisfying C1-C3, and different from $f(E_M)$, *i.e.*, there exists $E_M = E_{M1}$ such that $c(E_{M1}) \neq f(E_{M1})$. In this environment, the system reaches a steady state $(\hat{p}^*_1, \hat{r}^*_1)$ with $\hat{r}^*_1 = c(E_{M1}) \neq f(E_{M1})$. However, by Eq. S1.18 the optimal value for $\hat{r}^*$ in this environment is given by $f(E_{M1})$. So, the control law $c(E_M)$ does not drive the system to the optimal steady state in this environment, in contradiction with Condition C3.

S1.5.2 Precursor-only strategy

The precursor-only strategy is defined by:

$$\alpha = g(\hat{p}) = \frac{\hat{p}}{\hat{p} + \frac{K}{K+\hat{p}}(1+\hat{p})}. \tag{S1.23}$$

Here as well, C1 is satisfied by construction.

The ODE system under the control of g becomes

$$\begin{aligned} \frac{d\hat{p}}{d\hat{t}} &= (1-\hat{r})\, E_M - (1+\hat{p})\, \frac{\hat{p}}{K+\hat{p}}\, \hat{r}, \\ \frac{d\hat{r}}{d\hat{t}} &= (g(\hat{p}) - \hat{r})\ \frac{\hat{p}}{K+\hat{p}}\, \hat{r}. \end{aligned} \tag{S1.24}$$

The nullcline for $\hat{p}$ remains unchanged and is defined by

$$\frac{d\hat{p}}{dt} = 0 \Leftrightarrow \hat{r} = \frac{E_M}{E_M + \frac{\hat{p}}{K+\hat{p}}(1+\hat{p})}, \tag{S1.25}$$

while the nullcline for $\hat{r}$ is

$$\frac{d\hat{r}}{dt} = 0 \Leftrightarrow \begin{cases} \hat{p} = 0, \\ \hat{r} = 0, \\ \hat{r} = \frac{\hat{p}}{\hat{p} + \frac{K}{K+\hat{p}}(1+\hat{p})}. \end{cases} \tag{S1.26}$$

Hence, we also have a trivial unstable steady state at (0,1) (with zero growth). The second steady state is obtained from Eqs S1.25-S1.26:

$$\frac{E_M}{E_M + \frac{\hat{p}^*}{K+\hat{p}^*}(1+\hat{p}^*)} = \frac{\hat{p}^*}{\hat{p}^* + \frac{K}{K+\hat{p}^*}(1+\hat{p}^*)},$$

which we rearrange into

$$\hat{p}^* E_M + \frac{K}{K+\hat{p}^*}(1+\hat{p}^*)E_M = \hat{p}^* E_M + \frac{\hat{p}^*}{K+\hat{p}^*}(1+\hat{p}^*)\hat{p}^*.$$

This leads to

$$\hat{p}^* = \sqrt{KE_M},$$

and therefore

$$\hat{r}^* = g(\hat{p}^*) = \frac{\sqrt{KE_M}}{\sqrt{KE_M} + \frac{K}{K+\sqrt{KE_M}}(1+\sqrt{KE_M})} = \frac{E_M + \sqrt{KE_M}}{E_M + 2\sqrt{KE_M} + 1}.$$

From Eqs S1.17-S1.18, we recognize the optimal steady state for the environment E_M, validating Condition C3. We now look for the stability of this (optimal) steady state by deriving the Jacobian of this system:

$$J = \begin{pmatrix} -\frac{\hat{r}}{K+\hat{p}}\frac{\hat{p}^2+2K\hat{p}+K}{\hat{p}+K} & -E_M - \frac{\hat{p}}{K+\hat{p}}(1+\hat{p}) \\ \frac{\hat{r}}{K+\hat{p}}\left[\frac{K}{K+\hat{p}}(g(\hat{p})-\hat{r}) + \hat{p}K\frac{\hat{p}^2+2\hat{p}+K}{(\hat{p}^2+2K\hat{p}+K)^2}\right] & \frac{\hat{p}}{K+\hat{p}}(g(\hat{p})-2\hat{r}) \end{pmatrix}. \tag{S1.27}$$

Evaluated at $(\hat{p}^*, \hat{r}^*) = (\sqrt{KE_M}, g(\sqrt{KE_M}))$, the Jacobian becomes

$$J_{(\hat{p}^*,\hat{r}^*)} = \begin{pmatrix} -\frac{\sqrt{E_M}}{\sqrt{K}+\sqrt{E_M}} & -E_M - \frac{\sqrt{E_M}}{\sqrt{K}+\sqrt{E_M}}(1+\sqrt{KE_M}) \\ \frac{\sqrt{E_M}}{\sqrt{K}+\sqrt{E_M}}\frac{KE_M+2\sqrt{KE_M}+K}{K(E_M+2\sqrt{KE_M}+1)^2}g(\sqrt{KE_M}) & -\frac{\sqrt{E_M}}{\sqrt{K}+\sqrt{E_M}}g(\sqrt{KE_M}) \end{pmatrix}. \tag{S1.28}$$

Since K, E_M, and $g(\sqrt{KE_M}) > 0$, it follows immediately that the real part of the eigenvalues of this matrix are both negative. [2] Hence, the non-trivial steady state is stable, completing the proof of Condition C2.

Here again, it is interesting to observe that the expression in Eq. S1.23 is the only function $g(\hat{p})$ satisfying C1-C3. This can be proven in a similar way as for f.

S1.5.3 On-off strategy

The on-off strategy is defined by:

$$\alpha = h(\hat{p}, \hat{r}) = \begin{cases} 0, \text{ if } \hat{r} > g(\hat{p}), \\ 1, \text{ if } \hat{r} < g(\hat{p}), \\ \alpha^*_{opt}, \text{ if } (\hat{p}, \hat{r}) = (\hat{p}^*_{opt}, \hat{r}^*_{opt}). \end{cases} \tag{S1.29}$$

h is a static function of $\hat{p}$ and $\hat{r}$ (Condition C1).

As a consequence, the ODE system under the control of h is given by

$$\begin{aligned} \frac{d\hat{p}}{d\hat{t}} &= (1-\hat{r})\, E_M - (1+\hat{p})\, \frac{\hat{p}}{K+\hat{p}}\, \hat{r}, \\ \frac{d\hat{r}}{d\hat{t}} &= (h(\hat{p},\hat{r}) - \hat{r})\, \frac{\hat{p}}{K+\hat{p}}\, \hat{r}. \end{aligned} \tag{S1.30}$$

[2] Notice that the eigenvalues λ_1 and λ_2 of $J_{(\hat{p}^*,\hat{r}^*)}$ satisfy the inequalities $\mathrm{Tr}(J) = \lambda_1 + \lambda_2 < 0$ and $\det(J) = \lambda_1\lambda_2 > 0$.

Notice that the system has a discontinuitous right-hand side, due to the fact that α switches between 0 and 1 on $\hat{r} = g(\hat{p})$. Fig. S1.1 shows the dynamics of the system in the phase plane. Due to the direction of the vector fields relative to $\hat{r} = g(\hat{p})$, a *sliding mode* occurs on the latter curve [1]. The system is seen to evolve towards a locally asymptotically stable steady state, which is the single non-trivial steady state (Condition C2). This steady state coincides with the intersection of $\hat{r} = g(\hat{p})$ and the $\hat{p}$-nullcline, which is the steady state $(\hat{p}^*_{opt}, \hat{r}^*_{opt})$ allowing maximal growth, thus verifying Condition C3.

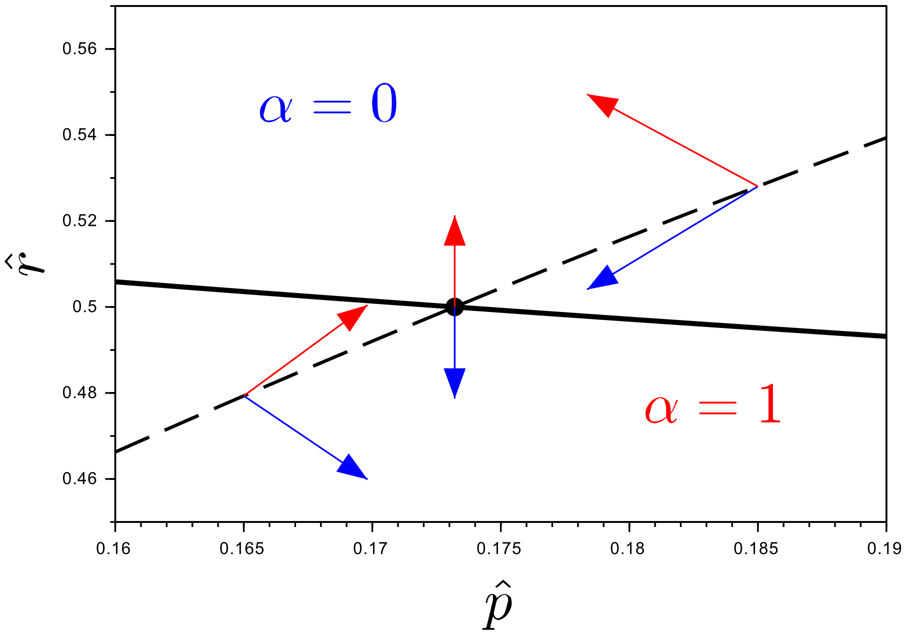

Figure S1.1: **Local stability of the on-off strategy.** The on-off strategy sets α to a value of 0 (1) when $\hat{r} > g(\hat{p})$ ($\hat{r} < g(\hat{p})$). The solid, black curve is the $\hat{p}$-nullcline. The dashed, black curve is the curve $\hat{r} = g(\hat{p})$. The arrows represent the vector fields for $\alpha = 0$ (in blue) and $\alpha = 1$ (in red). The intersection of the $\hat{p}$-nullcline and the curve $\hat{r} = g(\hat{p})$ corresponds to a unique non-trivial stable steady state, which is equal to $(\hat{p}^*_{opt}, \hat{r}^*_{opt})$ by Eq. S1.30.

References

[1] Filippov AF. Differential Equations with Discontinuous Righthand Sides. Dordrecht: Kluwer Academic Publishers; 1988. doi: 10.1007/978-94-015-7793-9.
