# Supplementary material for: Dynamical Allocation of Cellular Resources as an Optimal Control Problem: Novel Insights into Microbial Growth Strategies
Source: PLoS Comput Biol. 2016 Mar 9;12(3):e1004802. doi: 10.1371/journal.pcbi.1004802 (PMC4784908; doi:10.1371/journal.pcbi.1004802)
Supplement: S2 Text — (PDF) [file pcbi.1004802.s002.pdf]

S2 Text – Model parameters[1]

Nils Giordano [1,3], Francis Mairet [2], Jean-Luc Gouzé [2], Johannes Geiselmann [1,3,*], Hidde de Jong [3,*]

1 Université Grenoble Alpes, Laboratoire Interdisciplinaire de Physique (CNRS UMR 5588), 140 rue de la Physique BP 87, 38402 Saint Martin d'Hères, France
2 Inria, Sophia-Antipolis Méditerranée research centre, 2004 route des Lucioles, BP 93, 06902 Sophia-Antipolis Cedex, France
3 Inria, Grenoble - Rhône-Alpes research centre, 655 avenue de l'Europe, Montbonnot, 38334 Saint Ismier Cedex, France

*** Corresponding authors with equal contributions:**
Hidde.de-Jong@inria.fr, Hans.Geiselmann@ujf-grenoble.fr

Most of the conclusions of this paper are parameter-independent in the range of physically admissible values. The exact parameter values used in the simulations aim to represent relevant orders of magnitude. They were derived from the available literature on fast-growing bacteria (mostly *Escherichia coli*). This document describes this derivation for each parameter. In the *Methods* section of the main text, we also describe how some of them were validated by fitting the model to available experimental data (Fig. 3 in the main text).

The model parameters (Eqs. 3-4 in the main text) are listed in the table below:

Name	Unit	Description
e_M	h^{-1}	Constant characterizing nutrient composition of medium
k_R	h^{-1}	Rate constant of macromolecular synthesis
K_R	g L^{-1}	Half-saturation constant of macromolecular synthesis
β	L g^{-1}	Inverse of the cellular density of macromolecules
α	–	Resource allocation parameter

The values derived below are summarized in S1 Table.

e_M

By definition, e_M is the effective turnover of the metabolic macroreaction producing precursors from external substrates, obtained by dividing the reaction rate v_M by the enzyme concentration m (Eq. 6). The unit of e_M is min^{-1}, and can be decomposed as follows:

$$[e_M] = \frac{[\text{mass of metabolic product}]}{[\text{mass of enzyme M}] \cdot [\text{time}]} = \frac{1}{[\text{time}]}.$$

Note that $e_M = k_M\, s/(K_M + s)$ where k_M is a rate constant, indicating the maximal rate of conversion of external nutrients to precursor metabolites. e_M will thus vary with the concentration s of the external nutrients and the kind of nutrient. For example, the precursor mass that can be produced from 1 g of glucose is higher than that produced from 1 g of acetate.

How can we find a typical value for k_M, and thus for e_M (both have the same order of magnitude if we suppose that the reaction is not operating far below saturation, that is, $e_M \approx k_M$)? A reasonable estimate for k_M can be obtained from the turnover numbers of reactions

[1]Supporting Information of "Dynamical Allocation of Cellular Resources as an Optimal Control Problem: Novel Insights into Microbial Growth Strategies "

involved in the synthesis of charged tRNA, since the latter are directly consumed by the most abundant part of the gene expression machinery, the ribosomes.

Ref. [1] provides a typical value for such a reaction, catalyzed by glutaminyl-tRNA synthetase: $k_{cat,GlnRS} = 3.2\ \mathrm{s}^{-1}$, indicating that on average 3.2 glutaminyl-tRNA molecules are produced per glutaminyl-tRNA synthetase molecule per second. After conversion to mass units using molar weight from [2], this yields

$$k_{cat,GlnRS} = \frac{3.2 \cdot 147}{64.4 \cdot 10^3} \approx 10^{-3}\,\text{g of glutaminyl-tRNA} \cdot \text{g of enzyme}^{-1} \cdot \text{s}^{-1}.$$

We therefore take

$$k_M \approx 3.6\ \mathrm{h}^{-1},$$

and thus obtain an upper bound for e_M in our simulations.

k_R

k_R is the mass rate constant describing the maximal rate of conversion of precursors to macromolecules [h^{-1}]. As for e_M, we can decompose this into

$$[k_R] = \frac{[\text{mass of macromolecules}]}{[\text{mass of gene expression machinery}] \cdot [\text{time}]} = \frac{1}{[\text{time}]}$$

To obtain an order of magnitude for the mass of macromolecules, we focus on proteins since they are the most abundant macromolecules in the cell [3]. The dimensional analysis of k_R thus becomes:

$$\begin{aligned}[k_R] &= \frac{[\text{protein mass produced}]}{[\text{ribosomal mass}] \cdot [\text{hour}]} \\ &= \frac{[\text{moles of protein}] \cdot [\text{protein molar mass}]}{[\text{moles of ribosome}] \cdot [\text{ribosome molar mass}] \cdot [\text{hour}]} \\ &= \frac{[\text{moles of amino acids}] \cdot [\text{molar mass of amino acids}]}{[\text{moles of ribosome}] \cdot [\text{hour}] \cdot [\text{ribosome molar mass}]} \\ &\approx \frac{[\text{maximal protein elongation rate}] \cdot [\text{molar mass of amino-acids}]}{[\text{ribosome molar mass}]}.\end{aligned}$$

The values in the last equality are available from the literature [3, 4, 5, 6]. We obtain

$$k_R \approx \frac{10 \cdot 100}{10^6} \cdot 3600 \approx 3.6\ \mathrm{h}^{-1}.$$

This value is comparable with the translational capacity k_T, in μg of protein per μg of ribosomal protein per hour, given by Scott *et al.* [7]:

$$k_T = \frac{4.5\ \mu\text{g of protein} \ /\ \mu\text{g of RNA} \ /\ \text{h}}{0.76\ \mu\text{g of ribosomal protein} \ /\ \mu\text{g of RNA}} = 5.9\ \mathrm{h}^{-1}.$$

K_R

A value for the parameter K_R, representing the half-saturation constant of macromolecular synthesis, is more difficult to obtain from the literature. However, assuming that ribosomes operate close to saturation (80% over a range of growth rates [3]), we find that $K_R \approx 0.25\,p$, with p the total amino acid concentration. The total concentration of amino acids in the cell is around 150 mmol L^{-1} [8], which with a mean molecular weight of 118.9 g mol^{-1} for amino acids [5], yields a mass concentration of 17.8 g L^{-1}. These considerations led to the following order of magnitude for K_R:

$$K_R \approx 1\ \mathrm{g\ L}^{-1}.$$

β

β is the inverse of the cellular density of macromolecules, which has been shown constant during balanced growth over a large range of growth rates [9], and there is some data suggesting that β varies little during growth transitions as well [10]. From [11, 12] we take the following typical value for β:

$$\beta \approx \frac{1}{300} \approx 0.003 \text{ L g}^{-1}.$$

E_M and K

From the values of the parameter in the dimensional model, one can deduce the parameters in the nondimensional model used in the simulations:

$$E_M = \frac{e_M}{k_R} = \frac{3.6}{3.6} = 1 \quad , \quad K = \beta\, K_R = 3 \cdot 10^{-3} \cdot 1 = 0.003.$$

References

[1] Uter NT, Perona JJ. Long-range intramolecular signaling in a tRNA synthetase complex revealed by pre-steady-state kinetics. Proc Natl Acad Sci USA. 2004 Oct;101(40):14396–401. doi: 10.1073/pnas.0404017101.

[2] Freist W, Gauss DH, Ibba M, Söll D. Glutaminyl-tRNA synthetase. Biol Chem. 1997 Oct;378(10):1103–17.

[3] Ehrenberg M, Bremer H, Dennis PP. Medium-dependent control of the bacterial growth rate. Biochimie. 2013 Apr;95(4):643–58. doi: 10.1016/j.biochi.2012.11.012.

[4] Klumpp S, Scott M, Pedersen S, Hwa T. Molecular crowding limits translation and cell growth. Proc Natl Acad Sci USA. 2013 Oct;110(42):16754–9. doi: 10.1073/pnas.1310377110.

[5] Hachiya T, Terashima I, Noguchi K. Increase in respiratory cost at high growth temperature is attributed to high protein turnover cost in Petunia x hybrida petals. Plant Cell Environ. 2007 Oct;30(10):1269–83. doi: 10.1111/j.1365-3040.2007.01701.x.

[6] Yamamoto T, Izumi S, Gekko K. Mass spectrometry of hydrogen/deuterium exchange in 70S ribosomal proteins from *E. coli*. FEBS Lett. 2006 Jun;580(15):3638–42. doi: 10.1016/j.febslet.2006.05.049.

[7] Scott M, Gunderson CW, Mateescu EM, Zhang Z, Hwa T. Interdependence of cell growth and gene expression: origins and consequences. Science. 2010 Nov;330(6007):1099–102. doi: 10.1126/science.1192588.

[8] Bennett BD, Kimball EH, Gao M, Osterhout R, Van Dien SJ, Rabinowitz JD. Absolute metabolite concentrations and implied enzyme active site occupancy in *Escherichia coli*. Nat Chem Biol. 2009 Aug;5(8):593–9. doi: 10.1038/nchembio.186.

[9] Churchward G, Bremer H, Young R. Macromolecular composition of bacteria. J Theor Biol. 1982 Feb;94(3):651–70. doi: 10.1016/0022-5193(82)90305-8.

[10] Zhou Y, Vazquez A, Wise A, Warita T, Warita K, Bar-Joseph Z, et al. Carbon catabolite repression correlates with the maintenance of near invariant molecular crowding in proliferating *E. coli* cells. BMC Syst Biol. 2013 Dec;7:138. doi: 10.1186/1752-0509-7-138.

[11] Zimmerman SB, Trach SO. Estimation of macromolecule concentrations and excluded volume effects for the cytoplasm of *Escherichia coli*. J Mol Biol. 1991 Dec;222(3):599–620. doi: 10.1016/0022-2836(91)90499-V.

[12] McGuffee SR, Elcock AH. Diffusion, crowding & protein stability in a dynamic molecular model of the bacterial cytoplasm. PLoS Comput Biol. 2010 Mar;6(3):e1000694. doi: 10.1371/journal.pcbi.1000694.
