# Supplementary material for: Dynamical Allocation of Cellular Resources as an Optimal Control Problem: Novel Insights into Microbial Growth Strategies
Source: PLoS Comput Biol. 2016 Mar 9;12(3):e1004802. doi: 10.1371/journal.pcbi.1004802 (PMC4784908; doi:10.1371/journal.pcbi.1004802)
Supplement: S3 Text — (PDF) [file pcbi.1004802.s003.pdf]

S3 Text – Solution of optimal control problem[1]

Nils Giordano [1,3], Francis Mairet [2], Jean-Luc Gouzé [2], Johannes Geiselmann [1,3,*], Hidde de Jong [3,*]

1 Université Grenoble Alpes, Laboratoire Interdisciplinaire de Physique (CNRS UMR 5588), 140 rue de la Physique BP 87, 38402 Saint Martin d'Hères, France
2 Inria, Sophia-Antipolis Méditerranée research centre, 2004 route des Lucioles, BP 93, 06902 Sophia-Antipolis Cedex, France
3 Inria, Grenoble - Rhône-Alpes research centre, 655 avenue de l'Europe, Montbonnot, 38334 Saint Ismier Cedex, France

*** Corresponding authors with equal contributions:**
Hidde.de-Jong@inria.fr, Hans.Geiselmann@ujf-grenoble.fr

S3.1 Statement of the problem

We consider the dimensionless system defined by Eqs 11-12 in the main text, which are here repeated for clarity:

$$\begin{aligned} \frac{d\hat{p}}{d\hat{t}} &= (1-\hat{r})\,E_M - (1+\hat{p})\,\hat{r}\,\frac{\hat{p}}{K+\hat{p}}, \\ \frac{d\hat{r}}{d\hat{t}} &= \hat{r}\frac{\hat{p}}{K+\hat{p}}\,(\alpha(\hat{t})-\hat{r}). \end{aligned} \tag{S3.1}$$

As stated in the section *Biomass maximization as an optimal control problem* in the main text, the objective of this study is to maximize the growth rate on an interval $[0,\tau]$ after a nutrient upshift. With Eq. S1.8 in S1 Text, we have

$$\hat{\mu} = \hat{r}\,\frac{\hat{p}}{K+\hat{p}}.$$

In order to avoid boundary effects occurring over finite time intervals, notably the depletion of precursors just before τ, we solve the optimal control problem over an infinite horizon ($\tau \to \infty$). Consider the set of admissible controls

$$\mathcal{U} = \{\alpha : \mathbb{R} \to [0,1] \;\mid\; \alpha(\cdot) \text{ measurable}\}.$$

The optimization problem can then be stated as follows:

$$\alpha_{opt} = \arg\max_{\alpha\in\mathcal{U}} J(\alpha) \equiv \int_0^{+\infty} \hat{r}(\hat{t})\frac{\hat{p}(\hat{t})}{K+\hat{p}(\hat{t})}d\hat{t}, \tag{S3.2}$$

where $(\hat{p}(\hat{t}), \hat{r}(\hat{t}))$ is the unique solution of Eq. S3.1 starting at a given point $(\hat{p}_0, \hat{r}_0) \in \Omega \equiv \mathbb{R}_*^+ \times (0,1)$ for a given control $\alpha \in \mathcal{U}$.

Given that the performance index $J(\alpha)$ diverges, we actually consider *overtaking optimality* [1]. Consider the performance index of the trajectory $x(\cdot)$ emanating from x_0 and generated by $u(\cdot)$ defined for any $T \geq 0$ by

$$J_T(x_0, u(\cdot)) = \int_0^T f_0(x(t), u(t), t)dt.$$

[1]Supporting Information of "Dynamical Allocation of Cellular Resources as an Optimal Control Problem: Novel Insights into Microbial Growth Strategies "

A trajectory $x^*(\cdot)$ emanating from x_0 and generated by $u^*(\cdot)$ is said to be overtaking optimal if for any other trajectory $x(\cdot)$ emanating from x_0 and generated by $u(\cdot)$ the following holds

$$\liminf_{T\to\infty} \{J_T(x_0, u^*(\cdot)) - J_T(x_0, u(\cdot))\} \geq 0.$$

Roughly speaking, a trajectory is overtaking optimal if "the performance index catches up with the performance index of any other trajectory" [1].

S3.2 Maximum Principle

Necessary conditions on optimal trajectories can be obtained by the Infinite Horizon Maximum Principle [1]. Let $H(\hat{p}, \hat{r}, \lambda_p, \lambda_r, \lambda_0, \alpha)$ be the Hamiltonian of the system, defined by

$$H(\cdot) \equiv \lambda_p\, E_M\,(1-\hat{r}) - \hat{r}\,\frac{\hat{p}}{K+\hat{p}}\,[\lambda_p(1+\hat{p}) + \lambda_r\,\hat{r} + \lambda_0] + \alpha\,\lambda_r\,\hat{r}\,\frac{\hat{p}}{K+\hat{p}}.$$

Moreover, let α be an optimal control, and $\hat{x}(\cdot) = (\hat{p}(\cdot), \hat{r}(\cdot))$ the associated trajectory. Then, there exists $\lambda_0 \leq 0$ and an absolutely continuous map $\lambda = (\lambda_p, \lambda_r) : [0, +\infty) \to \mathbb{R}^2$ such that $(\lambda, \lambda_0) \neq 0$, and

$$\dot{\lambda}_p = -\frac{\partial H}{\partial \hat{p}} = \hat{r}\,\frac{K}{(K+\hat{p})^2}\,[\lambda_p\,(1+\hat{p}) + \lambda_r\,(\hat{r}-\alpha) + \lambda_0] + \hat{r}\,\frac{\hat{p}}{K+\hat{p}}\,\lambda_p, \tag{S3.3}$$

$$\dot{\lambda}_r = -\frac{\partial H}{\partial \hat{r}} = \lambda_p\,E_M + \frac{\hat{p}}{K+\hat{p}}\,[\lambda_p\,(1+\hat{p}) + \lambda_r\,(2\hat{r}-\alpha) + \lambda_0]\,. \tag{S3.4}$$

The maximization condition is given by:

$$\begin{aligned} &\alpha(\hat{t}) \in \arg\ max_{v\in[0,1]} H(\hat{x}(\hat{t}), \lambda(\hat{t}), \lambda_0, v), \\ &\text{almost everywhere on } [0, +\infty). \end{aligned} \tag{S3.5}$$

An extremal trajectory is a quadruplet $(\hat{x}(\cdot), \lambda(\cdot), \lambda_0, \alpha(\cdot))$ satisfying Eqs S3.1-S3.5. The extremal is said to be normal (resp. abnormal) if $\lambda_0 < 0$ (resp. $\lambda_0 = 0$). In the normal case, we normalize the adjoint vector so that $\lambda_0 = -1$.

From Eq. S3.5, it follows that the control strategy is given by the sign of the *switching function* $\phi(\cdot) \equiv \lambda_r\,\hat{r}\,\hat{p}/(K+\hat{p})$, that is,

$$\begin{cases} \alpha = 1 \iff \phi(\cdot) > 0, \\ \alpha = 0 \iff \phi(\cdot) < 0. \end{cases}$$

Finally, given that the system is autonomous, the Hamiltonian is conserved along any extremal trajectory.

S3.3 Characterization of singular arcs

Whenever ϕ is vanishing over a time interval, we say that the trajectory is *singular*. We will now characterize such trajectories. If $I = [\hat{t}_1, \hat{t}_2]$ is a singular arc, we have $\phi(\hat{t}) = \dot{\phi}(\hat{t}) = 0$, for all $\hat{t} \in [\hat{t}_1, \hat{t}_2]$, that is, $\lambda_r(\hat{t}) = 0$ and $\dot{\lambda}_r(\hat{t}) = 0$.

For abnormal extremal trajectories, we get $\lambda_p(\hat{t}) = 0$, in contradiction with the Maximum Principle, so there is no singular arc. An abnormal extremal trajectory is therefore a concatenation of bang arcs.

For normal extremal trajectories, using additionally that H is constant along an extremal trajectory, we obtain that λ_p is constant along a singular arc. By combining $\dot{\lambda}_p = 0$ and $\dot{\lambda}_r = 0$, we obtain $\hat{p}(\hat{t}) = \sqrt{E_M K} = \hat{p}^*_{opt}$. Using $d\hat{p}/d\hat{t} = 0$, we finally get $\hat{r}(\hat{t}) = \hat{r}^*_{opt}$. Thus, the singular arc is the optimal steady state, corresponding to a singular control $\alpha(\hat{t}) = \alpha^*_{opt}$, with α^*_{opt} depending on E_M (S1 Text).

A necessary condition of optimality for a singular arc is given by the Kelley condition [2]. We must differentiate ϕ with respect to $\hat{t}$ until α appears in the derivative. Along a singular arc, we obtain for $q = 2$:

$$(-1)^q \frac{\partial}{\partial \alpha} \frac{d^{2q}}{d\hat{t}^{2q}} \phi(\hat{t}) < 0,$$

satisfying the Kelley condition necessary for optimality. Given that the singular arc is of second order, an optimal trajectory can enter into the singular arc only by a *chattering arc* (also called the Fuller's phenomenon, *i.e.*, an arc with an infinite number of switches [2, 3]).

S3.4 Analysis of the adjoint system

Recalling that a switch corresponds to a change of sign of λ_r, the analysis of the adjoint system (Eqs S3.3-S3.4) may be useful to characterize the switches of extremal trajectories.

First, for the abnormal case, we can easily determine in the phase-plane the possible transitions between the four regions defined by the axes (see Fig. S3.1). A trajectory can cross at most twice the λ_p-axis, so we conclude that an abnormal extremal cannot have more than two switches. Thus, an abnormal extremal is a concatenation of at most three bang arcs ($\alpha(t) = 0$ or $\alpha(t) = 1$). When $\alpha(t) = 0$ or $\alpha(t) = 1$ for a long time, the growth rate tends to zero. We therefore conclude that abnormal extremal trajectories are not optimal.

Secondly, for the normal case, after the first switch, a trajectory with two consecutive switches in the regions $\{(\hat{p}, \hat{r}) \in \Omega \mid \hat{p} < \hat{p}^*_{opt}\}$ or $\{(\hat{p}, \hat{r}) \in \Omega \mid \hat{p} > \hat{p}^*_{opt}\}$ is not possible, as shown in Fig. S3.1. Therefore, such a trajectory is not optimal given that it does not fulfill the conditions given by the Maximum Principle. We conclude that if the optimal trajectory has a concatenation of bang arcs, the switches must alternatingly occur in the regions $\{(\hat{p}, \hat{r}) \in \Omega \mid \hat{p} < \hat{p}^*_{opt}\}$ and $\{(\hat{p}, \hat{r}) \in \Omega \mid \hat{p} > \hat{p}^*_{opt}\}$.

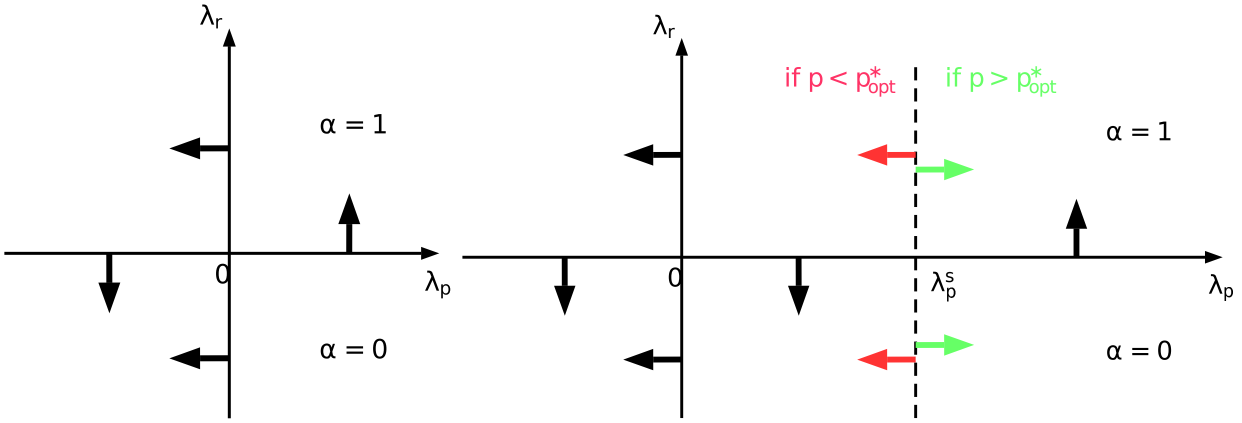

Figure S3.1: **Transitions between regions in the phase-plane for the adjoint system.** A switch occurs when a trajectory crosses the λ_p-axis. Left: abnormal case. An extremal trajectory cannot have more than two switches. Right: normal case. $(\lambda^s_p, 0)$ corresponds to the singular arc. After the first switch, an extremal trajectory cannot have two consecutive switches if it stays in the region $\{(\hat{p}, \hat{r}) \in \Omega \mid \hat{p} < \hat{p}^*_{opt}\}$ or $\{(\hat{p}, \hat{r}) \in \Omega \mid \hat{p} > \hat{p}^*_{opt}\}$.

S3.5 Optimal trajectories

From the Maximum Principle, we have shown that the optimal trajectory is a concatenation of bang arcs ($\alpha(t) = 0$ or $\alpha(t) = 1$) and possibly a singular arc corresponding to the optimal steady state $(\hat{p}(\hat{t}), \hat{r}(\hat{t})) = (\hat{p}^*_{opt}, \hat{r}^*_{opt})$. Moreover, if the optimal trajectory has a singular arc, it must enter it through a chattering arc (*i.e.*, with an infinite number of switches between $\alpha = 0$ and $\alpha = 1$).

These elements motivate the supposition that optimal solutions consist in a transient (chattering arc) towards the optimal steady state, after which they remain there (until the next change of environment). The chattering arc can be characterized by a switching curve $\hat{p} \mapsto \varphi(\hat{p})$ which passes through the optimal steady state. Defining A_0 and A_1 the regions above and below φ in the $(\hat{p}, \hat{r})$-plane, respectively, we conjecture that the following feedback control law is optimal:

$$\begin{cases} \alpha(\hat{t}) = 0 \text{ if } (\hat{p}(\hat{t}), \hat{r}(\hat{t})) \in A_0, \\ \alpha(\hat{t}) = 1 \text{ if } (\hat{p}(\hat{t}), \hat{r}(\hat{t})) \in A_1, \\ \alpha(\hat{t}) = \alpha_{opt} \text{ if } (\hat{p}(\hat{t}), \hat{r}(\hat{t})) = (\hat{p}^*_{opt}, \hat{r}^*_{opt}). \end{cases} \tag{S3.6}$$

Loosely speaking, the chattering arc corresponds to a spiral composed of bang arcs wrapping around the optimal steady state, where the switches alternatingly occur in the regions $\{(\hat{p}, \hat{r}) \in \Omega \mid \hat{p} < \hat{p}^*_{opt}\}$ and $\{(\hat{p}, \hat{r}) \in \Omega \mid \hat{p} > \hat{p}^*_{opt}\}$, in line with the analysis of the adjoint system. This is a first hint that the proposed control strategy is optimal. Moreover, our conjecture is also in line with the *turnpike property*: Trélat and Zuazua [4] have shown that, for quite a generic class of systems, the optimal strategy consists in staying at the optimal steady state (after a short transient).

As explained in the *Methods* section of the main text, we numerically solved the optimal control problem by the direct method using the `bocop` software [5]. It is important to stress that the optimization process was performed without any preliminary assumptions on the characteristics of the optimal trajectory. The fact that the numerical solution verifies the Maximum Principle (*i.e.*, the singular arc corresponds to the optimal steady state) and the Kelley condition (*i.e.*, the presence of a chattering arc) tends to confirm that the control strategy of Eq. S3.6 is optimal. As an aside, we note that due to the fact that numerical optimization was performed for a finite horizon, we actually obtained a second chattering arc escaping from the singular arc at the end of the simulation. This is a classical property of the turnpike strategy: the optimal trajectory leaves the optimal steady state just before the end of the time interval of interest, in our case consuming almost all precursors. This arc was removed from the plot in Fig. 4, because it does not occur with an infinite horizon and is therefore a numerical artifact for this study.

References

[1] Dean A. Carlson, Alain B. Haurie, and Arie Leizarowitz. *Infinite Horizon Optimal Control.* Springer, Berlin, Heidelberg, 1991.

[2] V. F. Borisov. Fuller's phenomenon: Review. *J. Math. Sci.*, 100(4):2311–54, July 2000.

[3] C. Marchal. Chattering arcs and chattering controls. *J. Optim. Theory Appl.*, 11(5):441–68, August 2013.

[4] Emmanuel Trélat and Enrique Zuazua. The turnpike property in finite-dimensional nonlinear optimal control. *J. Differ. Equ.*, 258(1):81–114, January 2015.

[5] Frédéric Bonnans, Pierre Martinon, and Vincent Grélard. Bocop - A collection of examples. Research report, 2012. eprint: https://hal.inria.fr/hal-00726992.
