# Supplementary material for: Dynamical Allocation of Cellular Resources as an Optimal Control Problem: Novel Insights into Microbial Growth Strategies
Source: PLoS Comput Biol. 2016 Mar 9;12(3):e1004802. doi: 10.1371/journal.pcbi.1004802 (PMC4784908; doi:10.1371/journal.pcbi.1004802)
Supplement: S4 Text — (PDF) [file pcbi.1004802.s004.pdf]

S4 Text – Kinetic model of the ppGpp system in *Escherichia coli*[1]

Nils Giordano [1,3], Francis Mairet [2], Jean-Luc Gouzé [2], Johannes Geiselmann [1,3,*], Hidde de Jong [3,*]

1 Université Grenoble Alpes, Laboratoire Interdisciplinaire de Physique (CNRS UMR 5588), 140 rue de la Physique BP 87, 38402 Saint Martin d'Hères, France
2 Inria, Sophia-Antipolis Méditerranée research centre, 2004 route des Lucioles, BP 93, 06902 Sophia-Antipolis Cedex, France
3 Inria, Grenoble - Rhône-Alpes research centre, 655 avenue de l'Europe, Montbonnot, 38334 Saint Ismier Cedex, France

*** Corresponding authors with equal contributions:**
Hidde.de-Jong@inria.fr, Hans.Geiselmann@ujf-grenoble.fr

The recently published model of Bosdriesz *et al.* [1] provides a synthesis of the currently available knowledge of the ppGpp regulatory system. Through the mechanisms of ppGpp production and degradation, it describes regulation of the synthesis of ribosomal RNA. We explain below how we use the model to compare the action of the ppGpp system with the on-off control strategy. The denomination of variables and parameters follows the Supporting Information of [1] , and is reproduced in Table S4.1 in order to make the text self-contained.

The evolution of the cellular concentration of ppGpp is described in [1] by

$$\frac{d\texttt{ppGpp}}{dt} = v_{RelA}(r_{t,tot}) + v_{spoT} - k_{spoT} \cdot \texttt{ppGpp}, \tag{S4.1}$$

where v_{spoT} and k_{spoT} are constants (see Table S4.1), and v_{RelA} is a function of $r_{t,tot}$, the total concentration of "stalled" ribosomes:

$$v_{RelA}(r_{t,tot}) = k_{RelA} \cdot RelA_{tot} \cdot \frac{r_{t,tot}}{K_{D,RelA} + r_{t,tot}}. \tag{S4.2}$$

The amount of stalled ribosomes is determined by the equilibrium between charged and uncharged tRNA, t_{ai} and t_i, in the cell:

$$r_{t,tot} = \sum_i r_{ti} = \sum_i r_i \frac{t_i/\kappa_t}{1 + t_{ai}/\kappa_{ta} + t_i/\kappa_t}, \tag{S4.3}$$

which can be rewritten as

$$r_{t,tot} = \sum_i r_i \frac{t_i/\kappa_t}{1 + (0.5r - t_i)/\kappa_{ta} + t_i/\kappa_t}, \tag{S4.4}$$

using the assumption that $t_{tot,i} = t_{ai} + t_i = 0.5 \cdot r$. r_i denotes the concentration of ribosomes recognizing amino acid i. Finally, with $r = \sum_i r_i$ the total ribosome concentration and a_i the concentration of amino acid i, the dynamics of the charged tRNA concentration is described by

$$\frac{dt_{ai}}{dt} = v_{tai}(a_i, t_i) - f_i \cdot v_{ribosome}(t_i, r), \tag{S4.5}$$

[1] Supporting Information of "Dynamical Allocation of Cellular Resources as an Optimal Control Problem: Novel Insights into Microbial Growth Strategies "

with $v_{tai}(a_i, t_i)$ the synthesis rate of charged tRNA, and $f_i \cdot v_{ribosome}(t_i, r)$ their consumption via protein synthesis. In particular,

$$v_{tai}(a_i, t_i) = k_{Si} \cdot S_{tot,i} \cdot \frac{t_i\, a_i}{t_i\, K_{Mai} + a_i\, K_{Mti} + t_i\, a_i}, \tag{S4.6}$$

$$v_{ribosome}(t_i, r) = k_{rib} \cdot r \cdot \left(1 + \sum_i \left[f_i \cdot \left(1 + \frac{t_i}{\kappa_t}\right) \frac{\kappa_{ta}}{0.5 \cdot r - t_i}\right]\right)^{-1}. \tag{S4.7}$$

For comparison with our framework, we need `ppGpp` as a direct function of the total amino acid concentration $a = \sum_i a_i$ (a proxy for precursors) and total ribosome concentration r (a proxy for gene expression machinery). To this end, we made two additionnal assumptions:

(A1) All concentrations specific to one type of amino acid i (a_i, t_{ai}, t_i, r_i) are in the same proportion $f_i = f = 1/20$ with respect to the total concentrations (a, t_a, t, r).

(A2) We apply a quasi-steady-state approximation (QSSA) to the dynamics of the concentration of the charged tRNAs (t_{ai}) and the concentration of ppGpp (`ppGpp`). That is, the dynamics of these variables are assumed fast relative to the dynamics of the amino acid concentrations (a_i) and the total ribosome concentration (r).

Using (A2), we can rewrite Eq. S4.5 as follow:

$$v_{tai}(a_i, t_i) = f_i \cdot v_{ribosome}(t_i, r), \tag{S4.8}$$

which, using (A1) and Eqs S4.6 and S4.7, leads to:

$$k_{Si} \cdot S_{tot,i} \cdot \frac{t_i\, a_i}{t_i\, K_{Mai} + a_i\, K_{Mti} + t_i\, a_i} = f_i \cdot k_{rib} \cdot r \cdot \left(1 + \frac{\kappa_{ta}}{0.5r - t_i} \cdot \left(1 + \frac{t_i}{\kappa_t}\right)\right)^{-1}. \tag{S4.9}$$

By rearranging both sides of the equation, t_i can be expressed as a function of a_i and r, which yields:

$$\begin{aligned}
&A{t_i}^2 + Bt_i + C = 0, \quad \text{with} \\
&A = \frac{k_{Si}\, S_{tot,i}\, a_i}{f_i\, k_{rib}\, r}\left(\frac{\kappa_{ta}}{\kappa_t} - 1\right) + K_{Mai} + a_i, \\
&B = \frac{k_{Si}\, S_{tot,i}\, a_i}{f_i\, k_{rib}\, r}(0.5\, r + \kappa_{ta}) + a_i\, K_{Mti} - 0.5\, r\, (K_{Mai} + a_i), \\
&C = -0.5\, r\, a_i\, K_{Mti},
\end{aligned} \tag{S4.10}$$

and therefore

$$t_i(a_i, r) = \frac{-B \pm \sqrt{B^2 - 4AC}}{2A}.$$

It is not difficult to show that the only solution on $[0, 0.5\, r]$ is

$$t_i(a_i, r) = \frac{-B + \sqrt{B^2 - 4AC}}{2A}. \tag{S4.11}$$

From this result, we obtain $r_{t,tot}$ as a function of a_i and r, by applying (A1) to Eq. S4.4:

$$r_{t,tot}(t_i, r) = r \cdot \frac{t_i/\kappa_t}{1 + (0.5r - t_i)/\kappa_{ta} + t_i/\kappa_t}, \tag{S4.12}$$

and substituting t_i by the expression of Eq. S4.11.

Finally, we apply (A2) to Eq. S4.1 and obtain the final expression giving the concentration of ppGpp as a function of the total amino acid and ribosome concentrations:

$$\texttt{ppGpp}(a_i, r) = \frac{1}{k_{spoT}} \left(k_{RelA} \cdot RelA_{tot} \cdot \frac{r_{t,tot}(a_i, r)}{K_{D,RelA} + r_{t,tot}(a_i, r)} + v_{spoT} \right). \tag{S4.13}$$

This function is represented in Fig. S4.1 with parameters taken from Table S4.1.

The plotted surface of the function resembles the inverse of the on-off control strategy in Fig. 8, as expected, bearing in mind that ppGpp has an inhibitory effect on the synthesis of ribosomal RNA. We assumed a Michaelis-Menten inhibition for the regulatory effect of ppGpp on rRNA synthesis, and thus indirectly on the synthesis of ribosomal proteins [2, 3]:

$$\alpha(\texttt{ppGpp}) = \frac{K_I}{K_I + \texttt{ppGpp}}. \tag{S4.14}$$

The inhibitory constant K_I lies in the dynamical range of variation of ppGpp. In Fig. 8 in the main text, we took $K_I = 10\ \mu$M.

References

[1] Bosdriesz E, Molenaar D, Teusink B, Bruggeman FJ. How fast-growing bacteria robustly tune their ribosome concentration to approximate growth-rate maximization. FEBS J. 2015 Mar;282:2029–204. doi: 10.1111/febs.13258.

[2] Potrykus K, Cashel M. (p)ppGpp: still magical? Annu Rev Microbiol. 2008;62:35–51. doi: 10.1146/annurev.micro.62.081307.162903.

[3] Keener J, Nomura M. Regulation of ribosome synthesis. In: Neidhardt FC, III RC, Ingraham JL, Lin ECC, Low KB, Magasanik B, et al., editors. *Escherichia coli* and *Salmonella*: Cellular and Molecular Biology. 2nd ed. Washington, DC: ASM Press; 1996. p. 1417–31.

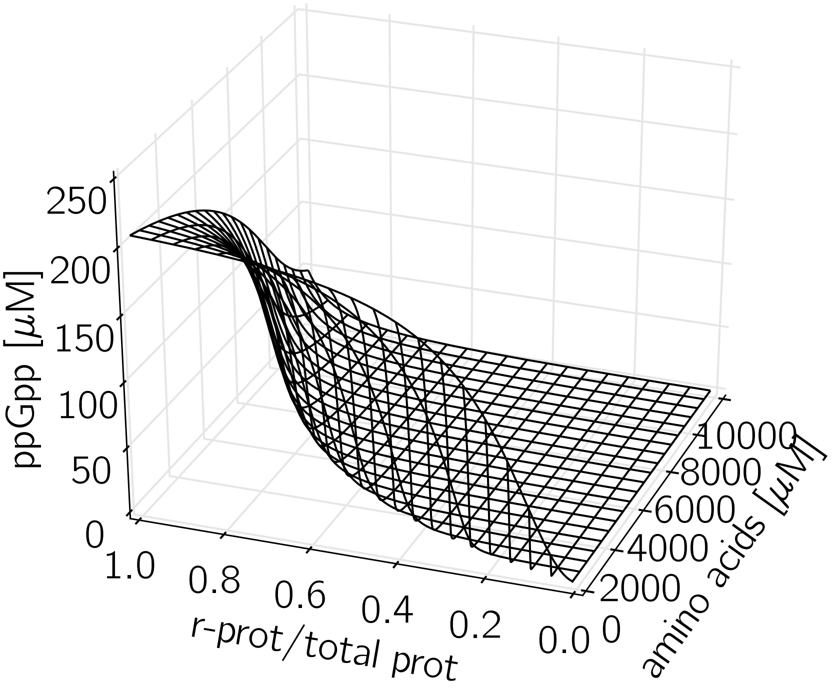

Figure S4.1: **ppGpp concentration is a function of total ribosome and amino acid concentrations.**
We assume the dynamics of ppGpp to be fast on the time-scale of changes in the ribosome and amino acid concentrations. The concentration of ppGpp can thus be expressed as a function of the latter two variables, using the model of Bosdriesz *et al.* [1]. Parameters are taken from Table S4.1.

Symbol	Value	Unit	Description
a_i	–	μM	Concentration of aa i (not incorporated in protein)
t_{ai}	–	μM	Concentration of tRNA charged with aa i
t_i	–	μM	Concentration of free tRNA conjugate to aa i
$t_{tot,i}$	$0.5 \cdot r$	μM	Total concentration of tRNA conjugate to aa i
r_i	–	μM	Total concentration of ribosome with an A-site for aa i
r_{ti}	–	μM	Ribosomes with uncharged tRNA in an A-site for aa i
ppGpp	–	μM	Concentration of ppGpp
a	$\sum_i a_i$	μM	Total concentration of aa (not incorporated in protein)
t_a	$\sum_i t_{ai}$	μM	Total concentration of tRNA charged with aa
t	$\sum_i t_i$	μM	Total concentration of free tRNA
$r_{t,tot}$	$\sum_i r_{ti}$	μM	Total concentration of uncharged tRNA bound to ribosomes
r	$\sum_i r_i$	μM	Total concentration of ribosomes
v_{RelA}	–	μM/s	Rate of RelA-catalyzed ppGpp synthesis
v_{SpoT}	10^{-3}	μM/s	Rate of ppGpp synthesis by SpoT
v_{tai}	–	μM/s	Rate of amino-acyl tRNA i synthetase
$v_{ribosome}$	–	μM/s	Total rate of protein synthesis
k_{rib}	20	s^{-1}	k_{cat} of protein elongation
k_{RelA}	75	s^{-1}	k_{cat} of ppGpp synthesis by RelA
$K_{D,RelA}$	0.26	μM	Michaelis constant of RelA-catalyzed ppGpp production
$RelA_{tot}$	1/15	μM	RelA concentration
k_{SpoT}	ln(2)/30	s^{-1}	Rate of ppGpp degradation by SpoT
κ_t	500	μM	Dissociation constant of uncharged tRNA-ribosome complex
κ_{ta}	1	μM	Dissociation constant of charged tRNA-ribosome complex
k_{Si}	100	s^{-1}	k_{cat} of aminoacyl-tRNA synthetase
$S_{tot,i}$	1	μM	Total concentration of aminoacyl-tRNA synthetase for aa i
K_{Mai}	100	μM	Michaelis constant of aa-tRNA synthetase for amino acids
K_{Mti}	1	μM	Michaelis constant of aa-tRNA synthetase for uncharged tRNA
f_i	1/20	–	Proportion of aa i in proteins (Assumption A1)

Table S4.1: **Parameters and variables reused from Bosdriesz *et al.* [1].** The abbreviation aa denotes amino acids.
