# Supplementary material for: Dynamical Allocation of Cellular Resources as an Optimal Control Problem: Novel Insights into Microbial Growth Strategies
Source: PLoS Comput Biol. 2016 Mar 9;12(3):e1004802. doi: 10.1371/journal.pcbi.1004802 (PMC4784908; doi:10.1371/journal.pcbi.1004802)
Supplement: S1 Table — (PDF) [file pcbi.1004802.s005.pdf]

S1 Table – Parameter values of self-replicator model[1]

Nils Giordano [1,3], Francis Mairet [2], Jean-Luc Gouzé [2], Johannes Geiselmann [1,3,*], Hidde de Jong [3,*]

1 Université Grenoble Alpes, Laboratoire Interdisciplinaire de Physique (CNRS UMR 5588), 140 rue de la Physique BP 87, 38402 Saint Martin d'Hères, France
2 Inria, Sophia-Antipolis Méditerranée research centre, 2004 route des Lucioles, BP 93, 06902 Sophia-Antipolis Cedex, France
3 Inria, Grenoble - Rhône-Alpes research centre, 655 avenue de l'Europe, Montbonnot, 38334 Saint Ismier Cedex, France

*** Corresponding authors with equal contributions:**
Hidde.de-Jong@inria.fr, Hans.Geiselmann@ujf-grenoble.fr

Parameter	Unit	Literature value	Fitted value
γ	–	No value	1.39
k_R	h^{-1}	3.6	2.23
e_M for M63+glycerol	h^{-1}	< 3.6	0.587
e_M for M63+glucose	h^{-1}	< 3.6	0.867
e_M for cAA+glycerol	h^{-1}	< 3.6	1.07
e_M for cAA+glucose	h^{-1}	< 3.6	1.57
e_M for RDM+glycerol	h^{-1}	< 3.6	3.48
e_M for RDM+glucose	h^{-1}	< 3.6	4.76
βK_R	–	0.003	Not fitted

Table S1: **Parameter values of self-replicator model** The parameter values in the model were obtained by fitting Eq. 25 to the data of Scott et al [1] (Fig. 3 in the main text), as described in the *Methods* section. They are compared with order-of-magnitude estimates from the literature (S2 Text).

References

[1] Scott M, Gunderson CW, Mateescu EM, Zhang Z, Hwa T. Interdependence of cell growth and gene expression: origins and consequences. Science. 2010 Nov;330(6007):1099–102. doi: 10.1126/science.1192588.

[1] Supporting Information of "Dynamical Allocation of Cellular Resources as an Optimal Control Problem: Novel Insights into Microbial Growth Strategies "
