# Supplementary material for: Dynamical Allocation of Cellular Resources as an Optimal Control Problem: Novel Insights into Microbial Growth Strategies
Source: PLoS Comput Biol. 2016 Mar 9;12(3):e1004802. doi: 10.1371/journal.pcbi.1004802 (PMC4784908; doi:10.1371/journal.pcbi.1004802)
Supplement: S1 Fig — (PDF) [file pcbi.1004802.s006.pdf]

S1 Figure – Simple control strategies for the self-replicator of bacterial growth[1]

Nils Giordano [1,3], Francis Mairet [2], Jean-Luc Gouzé [2], Johannes Geiselmann [1,3,*], Hidde de Jong [3,*]

1 Université Grenoble Alpes, Laboratoire Interdisciplinaire de Physique (CNRS UMR 5588), 140 rue de la Physique BP 87, 38402 Saint Martin d'Hères, France
2 Inria, Sophia-Antipolis Méditerranée research centre, 2004 route des Lucioles, BP 93, 06902 Sophia-Antipolis Cedex, France
3 Inria, Grenoble - Rhône-Alpes research centre, 655 avenue de l'Europe, Montbonnot, 38334 Saint Ismier Cedex, France

*** Corresponding authors with equal contributions:**
Hidde.de-Jong@inria.fr, Hans.Geiselmann@ujf-grenoble.fr

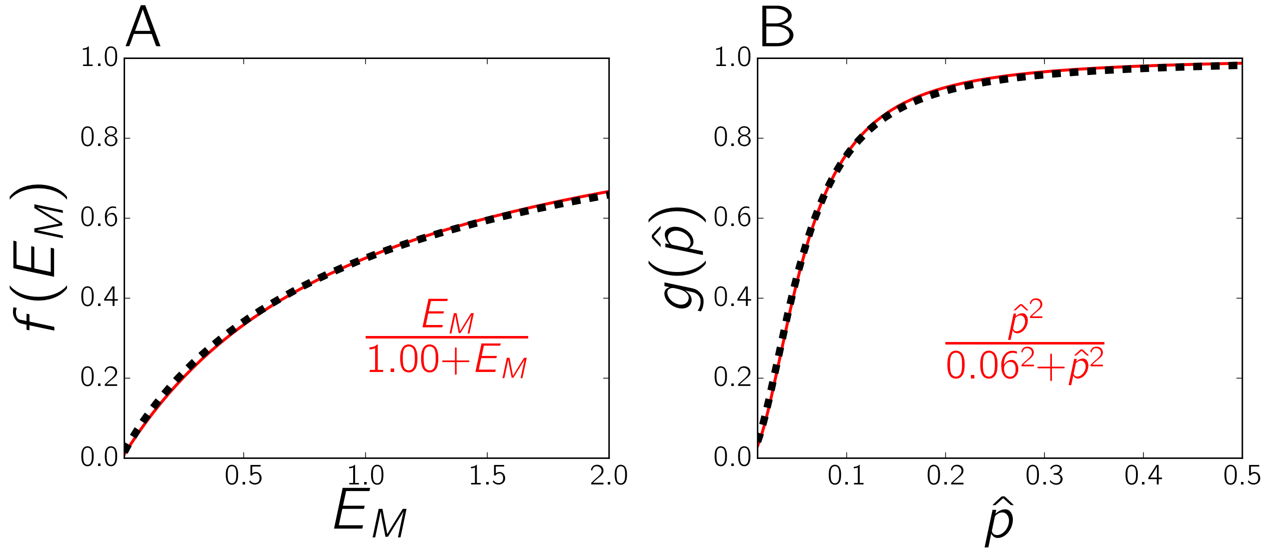

Figure S1: *A:* Nutrient-only strategy: $\alpha = f(E_M)$. The dashed, black curve is the (unique) strategy driving the system exactly to the optimal steady state, that is, the state in which growth occurs at the maximal rate supported by E_M. The function f is defined by Eq. 27 in the *Methods* section of the main text. The solid, red curve is an approximation of this function by the simple Michaelis-Menten curve of Eq. 16, with $K_{mE} = 1.0$. *B:* Precursor-only strategy: $\alpha = g(\hat{p})$. The dashed, black curve is the (unique) strategy driving the system exactly to the optimal steady state. The function g is defined by Eq. 28 in the *Methods* section of the main text. The solid, red curve is an approximation of this function by the simple Hill curve of Eq. 18, with $K_{mp} = 0.06$ and a cooperativity coefficient 2.

[1]Supporting Information of "Dynamical Allocation of Cellular Resources as an Optimal Control Problem: Novel Insights into Microbial Growth Strategies "
